# Direct Synthesis of Polyaromatic Cyclophanes Containing Bis-Methylene-Interrupted *Z*-Double Bonds and Study of Their Antitumor Activity In Vitro

**DOI:** 10.3390/ijms22168787

**Published:** 2021-08-16

**Authors:** Vladimir A. D’yakonov, Ilgiz I. Islamov, Lilya U. Dzhemileva, Elina Kh. Makarova, Usein M. Dzhemilev

**Affiliations:** Institute of Petrochemistry and Catalysis, Russian Academy of Sciences, Pr. Oktyabrya 141, 450075 Ufa, Russia; iislamovi@gmail.com (I.I.I.); makarovaelina87@gmail.com (E.K.M.); dzhemilev@anrb.ru (U.M.D.)

**Keywords:** cyclophanes, 1,5-dienoic compounds, 1,2-dienes, cyclomagnesiation, homogeneous catalysis

## Abstract

An original synthetic route was developed for the preparation of previously unknown unsaturated polyaromatic macrolactones containing a 1Z,5Z-diene moiety in 48–71% yields and with >98% stereoselectivity. The method is based on intermolecular cyclocondensation of aromatic dicarboxylic acids with α,ω-alka-nZ,(n+4)Z-dienediols (1,12-dodeca-4Z,8Z-dienediol, 1,14-tetradeca-5Z,9Z-dienediol, 1,18-octadeca-7Z,11Z-dienediol) mediated by *N*-(3-dimethylaminopropyl)-*N*′-ethylcarbodiimide hydrochloride (EDC)/4-dimethylaminopyridine (DMAP). The unsaturated diols were prepared by successive homo-cyclomagnesiation of tetrahydropyran ethers of O-containing 1,2-dienes with EtMgBr in the presence of Mg metal and the Cp_2_TiCl_2_ catalyst (10 mol.%) and subsequent treatment with 0.1 equiv. of para-toluenesulfonic acid of pyran ethers formed after the acid hydrolysis of magnesacyclopentanes. The resulting cyclophanes exhibited high cytotoxic activity in vitro against Jurkat, K562, U937, and HL60 cancer lines. Additionally, the synthesized products were studied for their effect on mitochondria, ability to induce apoptosis, and influence on the cell cycle using modern flow cytometry methods.

## 1. Introduction

The last 15–20 years have seen considerable interest in cyclophanes, which are bridged aromatic macrocyclic compounds composed of aromatic or heteroaromatic rings connected by aliphatic chains. This type of macrolide is widely used in various fields, including supramolecular chemistry, materials science, catalysis, and pharmaceutics. Owing to their unique structural features, cyclophanes are employed for the construction of cage structures, recognition of molecules and receptors, and as building blocks for organic catalysts for the preparation of crown ethers and cryptands [1,2].

The cyclophane skeleton is the key structural unit of many biologically active natural compounds, for example, acetylene-containing cyclophane Nostocyclyne A, isolated from *Nostoc* sp., exhibits antimicrobial activity against *S. aureus* and *Bacillus subtilis* [3]. Turriane-type cyclophanes are efficient agents for the cleavage of supercoiled DNA; they inhibit acetylcholinesterase (AChE) and exhibit high cytotoxic activity against KB and L1210 tumor cell lines [4,5,6].

Haouamine A isolated from *Aplidium haouarianum* was studied for activity against A-549 human lung carcinoma cells, HT-29 and HCT-116 human colon cancer cells, and PC-3 human prostate cancer cells. High activity and selectivity of the macrolide against the HT-29 cell line was found (IC_50_ = 0.1 µg/mL) [7,8].

The cyclophane derivative Engelhardione is considered as a potential antituberculosis drug, exhibiting clear-cut antibacterial properties against *M. tuberculosis*, *E. faecalis*, and *S. aureus* [9]. Water-soluble HIV protease inhibitors were synthesized on the basis of para-cyclophanes [10].

Macrocyclic natural bis-dibenzyl compounds, for example, riccardins C, D, and H, marchantins A and B, cavicularin, etc., are used as potent antifungal and antibacterial agents and also exhibit cytotoxic activity against P-388 lymphocytic leukemia and nasopharyngeal carcinoma cells [11,12,13].

**Figure 1:**
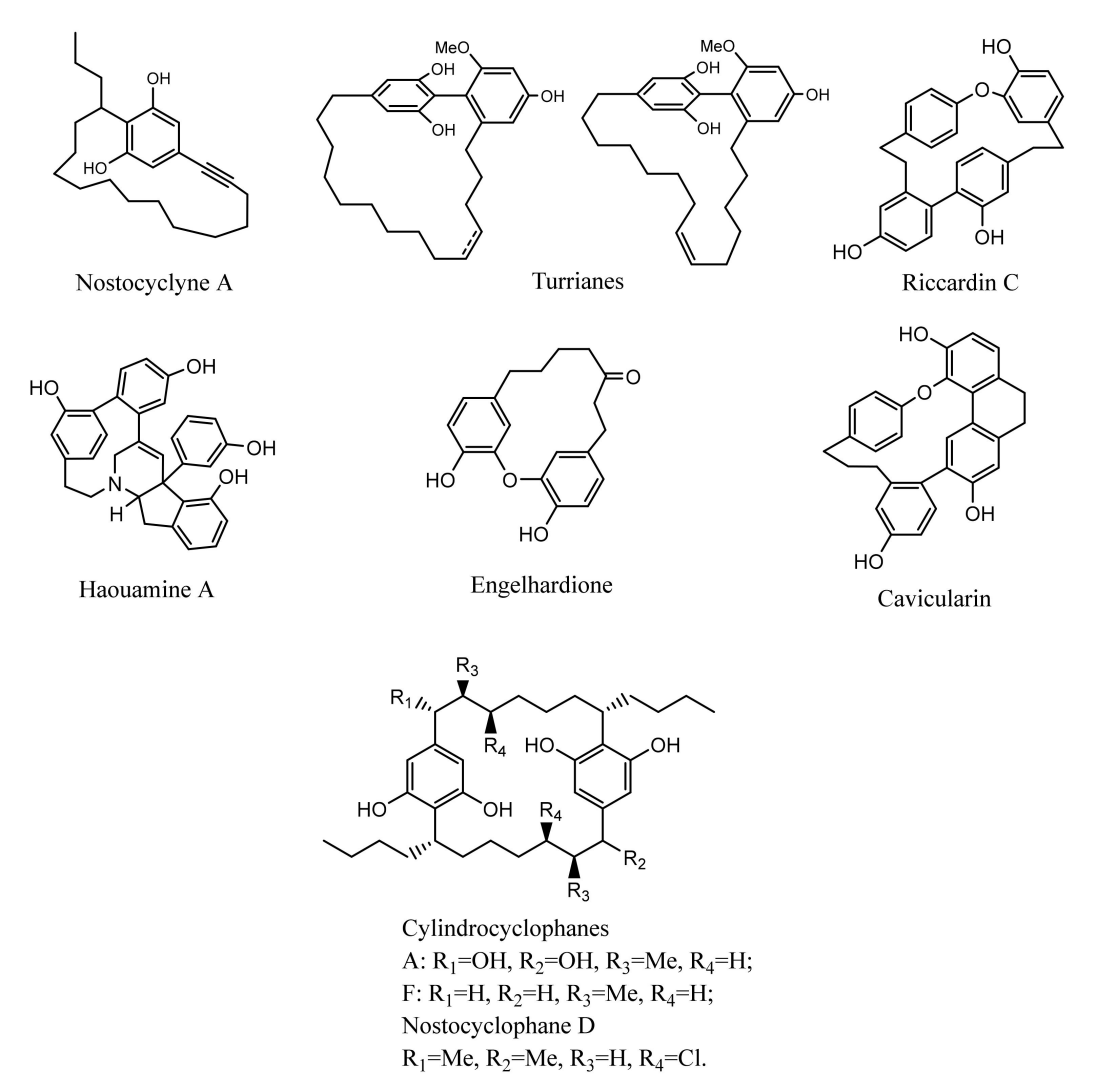
Structures of some natural cyclophanes.

The paracyclophane derivatives (-)-cylindrocyclophanes A and F and nostocyclophane D isolated from the blue-green algae *Cylindrospermum licheniforme* show high cytotoxic activities (IC_50_ = 2–10 μg/mL) against KB and LoVo tumor cell lines [14,15] (Figure 1).

**Figure 2:**
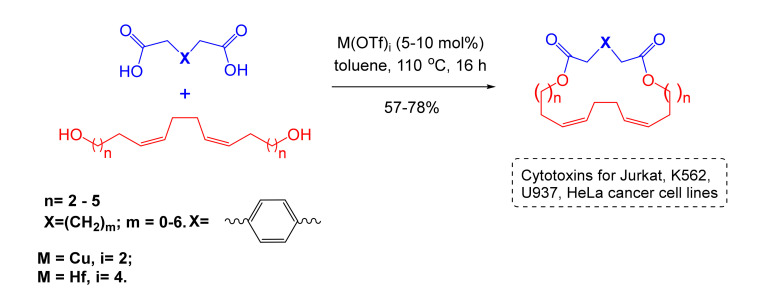
Previously developed synthesis of macrodiolides.

Recently, we developed an efficient method for the synthesis of previously unknown macrodiolides containing 1*Z*,5*Z*-diene groups in the molecule (Figure 2) [16,17]. The unsaturated macrodiolides that we synthesized showed high cytotoxic activity in vitro against the Jurkat, K562, U937, Hek293, and HeLa cell lines, and, in particular, they were more active than their saturated analogues. The macrocycles containing an aromatic ring in the molecule exhibited selective cytotoxic activity against the Jurkat and U937 cell lines (IC_50_ = 0.02–0.06 µM) [17,18]. Therefore, it was of interest to find out how the cytotoxicity of 1*Z*,5*Z*-diene compounds changes in the presence of several aromatic moieties.

In view of the foregoing and to pursue our studies on the development of stereoselective syntheses of biologically active compounds containing 1*Z*,5*Z*-diene moieties [19,20,21,22,23,24,25,26,27,28], here we report the synthesis of new macrolides by catalytic intermolecular esterification of α,ω-alka-n*Z*,(n+4)*Z*-dienediols with polyaromatic dicarboxylic acids and study of the obtained compounds for cytotoxic activity against various tumor cell lines.

## 2. Results

### 2.1. Chemistry

The strategy towards the target macrodiolides includes the successive synthesis of the above-mentioned α,ω-alka-nZ,(n+4)Z-dienediols by the Ti-catalyzed intermolecular homo-cyclomagnesiation of O-containing 1,2-dienes with Grignard reagents, which we developed previously, and intermolecular esterification of the products with aromatic dicarboxylic acids. 

**Scheme 1:**
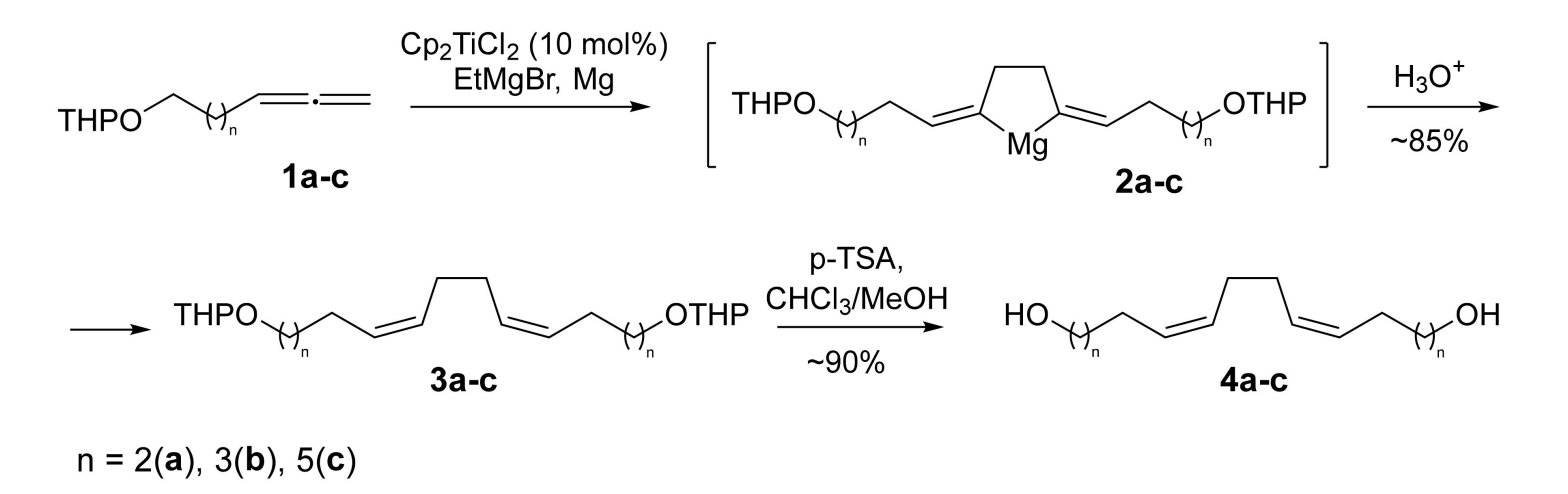
Synthesis of α,ω-alca-nZ,(n+4)Z-dienediols using Ti-catalyzed intermolecular cyclomagnesiation of 1,2-dienes.

Implementation of this strategy for the synthesis of unsaturated macrocyclic compounds was started with the preparation of α,ω-alka-nZ,(n+4)Z-dienediols (1,12-dodeca-4Z,8Z-dienediol (**4a**), 1,14-tetradeca-5Z,9Z-dienediol (**4b**), and 1,18-octadeca-7Z,11Z-dienediol (**4c**)). The intermolecular homo-cyclomagnesiation of the tetrahydropyran ethers of alkadienols **1a**–**c** (hexa-4,5-dienol (**1a**), hepta-5,6-dienol (**1b**), and nona-7,8-dienol (**1c**)) with EtMgBr in the presence of magnesium metal and the Cp_2_TiCl_2_ catalyst (**1**:EtMgBr:Mg:[Ti] = 10:20:24:0.5; Et_2_O, 6 h, 20–22 °C) afforded magnesacyclopentanes **2a**–**c**, which were subjected to acid hydrolysis to give bis-tetrahydropyran ethers **3a**–**c** in ~85–92% yields and with >98% stereoselectivity (Scheme 1). As the final step of the synthesis, compounds **3a**–**c** were treated with 0.1 equiv. of para-toluenesulfonic acid in a methanol–chloroform mixture (1:1), which afforded the target α,ω-alka-nZ,(n+4)Z-dienediols **4a**–**c** in good yields after 2 h at 55 °C.

The aromatic dicarboxylic acids were synthesized from methyl salicylate and dibromoxylenes (α,α′-dibromo-o-xylene, α,α′-dibromo-m-xylene, α,α′-dibromo-p-xylene) in two steps by the previously described procedure [29].

Initially, for the synthesis of target cyclophanes, the well-proven method of intermolecular macrolactonization in the presence of catalytic amounts of transition metal triflates was employed [30,31]. However, this method led to the preparation of cyclophanes in very low yields (less than 20%). Changing the reaction conditions (the use of other solvents, the increase in reaction time, variation of the amount of catalyst Hf(OTf)_4_ in the range of 5–20%, and temperature change of the reaction) did not lead to a significant increase in the yields of the target compounds. Therefore, to implement our plans, alternative methods of macrocyclization using carbodiimides were tested. This strategy turned out to be successful. As a result, we synthesized new cyclophans containing a 1Z,5Z-diene moiety in the structure.

It has been shown that α,ω-alka-nZ,(n+4)Z-dienediols (1,12-dodeca-4Z,8Z-dienediol (**4a**), 1,14-tetradeca-5Z,9Z-dienediol (**4b**), 1,18-octadeca-7Z,11Z-dienediol (**4c**)), and aromatic dicarboxylic acids (**5a**–**c**) can be involved in intermolecular cyclocondensation mediated by DMAP and EDC·HCl [31,32].

**Scheme 2:**
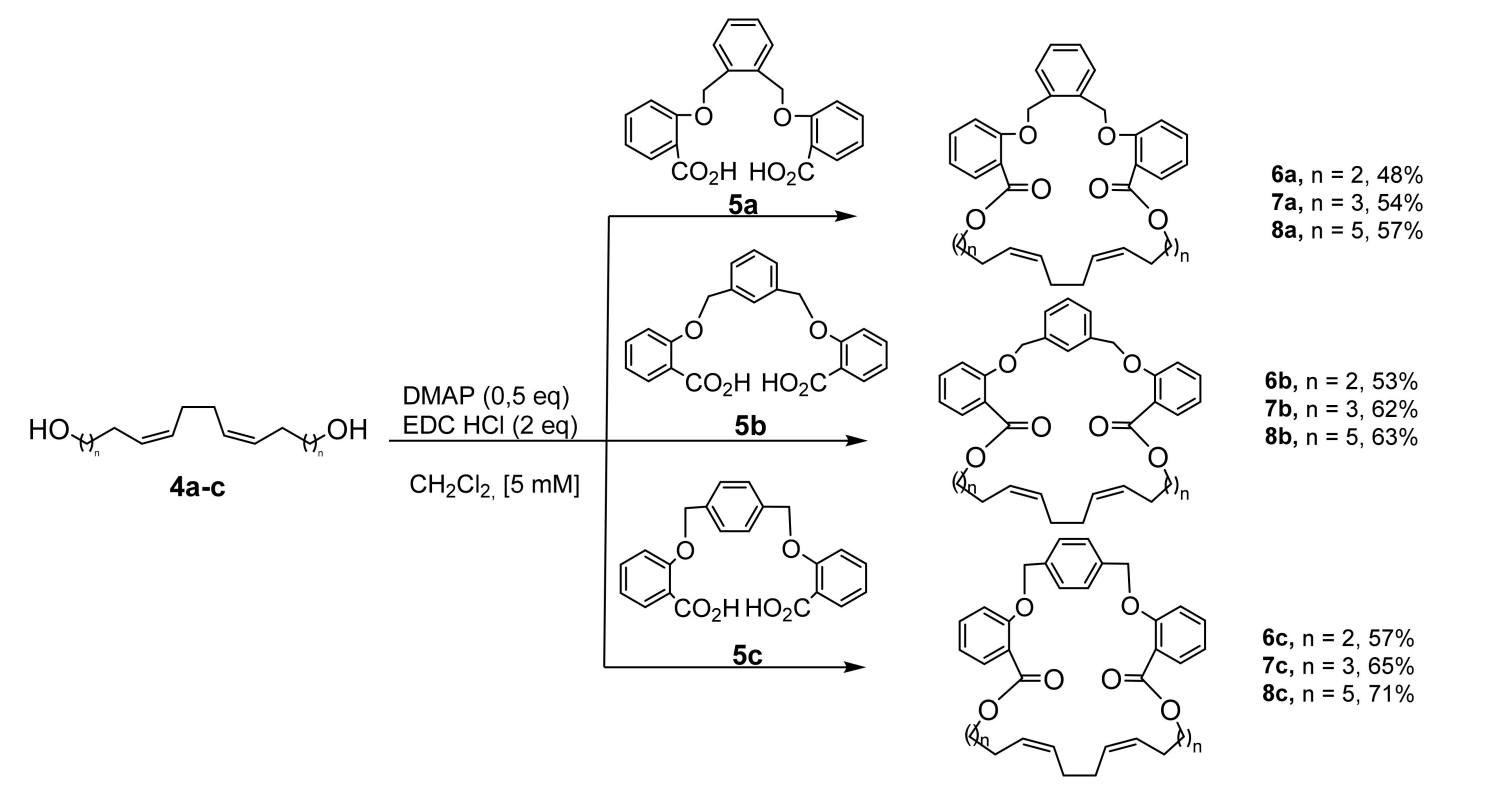
EDC·HCl/DMAP-mediated intermolecular cyclocondensation of a, ω-alka-nZ,(n+4)Z-dienols with aromatic dicarboxylic acids.

Initially, we attempted to affect the cyclization of 1,12-dodeca-4Z,8Z-dienediol (**4a**) with dicarboxylic acid (**5a**) prepared from methyl salicylate and o-dibromoxylene. The highest yield was attained for the diol (**4a**):dicarboxylic acid (**5a**):DMAP:EDCI ratio of 1:1:0.5:2 in dichloromethane with reactant concentrations of [5 mM]. Under these conditions, 1:1 cycloadduct was formed as the only product in a 48% yield. The macrocyclization of diol (**4a**) with dicarboxylic acids prepared from methyl salicylate and m-dibromoxylene (**5b**), and from methyl salicylate and p-dibromoxylene (**5c**) carried out under the same conditions furnished the target macrolactones in 53% and 57% yields, respectively. As in the previous example, only 1:1 adducts resulting from cyclocondensation were obtained (Scheme 2).

The macrocyclization reactions carried out between 1,14-tetradeca-5Z,9Z-dienediol (**4b**) and aromatic acids (**5a**–**c**) afforded the corresponding macrolactones in 54–65% yields. The elucidated regularities of macrolide synthesis were also retained when the reaction was performed for 1,18-octadeca-7Z,11Z-dienediol (**4c**). It is noteworthy that the yields of the products increased along the series of dicarboxylic acids from **5a** to **5c** and also with the increasing hydrocarbon chain length of the diols from **4a** to **4c**. As a result, the reaction of 1,18-octadeca-7Z,11Z-dienediol (**4c**) with dicarboxylic acid **5c** afforded the target cyclophane in an overall yield of 71% (Scheme 2).

The structure of the prepared macrocycles **6**–**8** was established by mass spectrometry and ^1^H and ^13^C NMR spectroscopy, using 2-D heteronuclear correlation experiments (HSQC, HMBC). Please see the Supplementary Materials.

### 2.2. Biological Evaluation

#### 2.2.1. The In Vitro Antitumor Activity of Cyclophanes (**6**–**8**)

The cytotoxicity of the prepared compounds was studied using Jurkat, K562, U937, and HL-60 suspension tumor cells; conditionally normal Hek293 cells; and normal fibroblasts. The IC_50_ values were determined by flow cytofluorimetry with the Guava ViaCount reagent kit (Millipore, Bedford, MA, USA).

Analysis of the results (Table 1) showed that the highest activity is inherent in derivatives of dicarboxylic acids **5a**–**c** with 1,18-octadeca-7Z,11Z-dienediol (**4c**). In each group of compounds, the cytotoxic effect against any type of tested cell tends to decrease in the series meta- < ortho- < para-, irrespective of the chain length of dienediol **4a**–**c** (Table 1). The cytotoxicity decreases in the series of cell lines K562 > U937 > HL-60 > Jurkat, with the above-mentioned dependence on the cyclophane structure being maintained. In addition, comparing the results on the cytotoxicity of the obtained macrodiolides within the framework of this study with the cytotoxicity of the previously synthesized macrodiolides with alkyl chains with different numbers of methylene units [16,17,18], we found that the introduction of polyaromatic chains does not lead to a significant change in the cytotoxicity of the synthesized macrodiolides. 

Furthermore, it can be seen that the selectivity index (SI) of inhibition for the Jurkat, K562, U937, and HL-60 cancer cells is 3–5 times higher than for the human embryonic kidney cells (Hek293) and 7–12 times higher than for normal fibroblasts.

#### 2.2.2. Induction of Apoptosis

We studied the new macrodiolides for the induction of apoptosis and for the effect on the cell cycle. For this purpose, we chose the Jurkat, K562, and U937 cell lines. Apoptosis was studied by flow cytofluorimetry using Annexin V and 7-aminoactinomycin D (7AAD) (Guava Nexin reagent (Millipore, Bedford, MA, USA)). After incubation, the cells were analyzed using a NovoCyte™ 2000 Flow Cytometry System (ACEA). Apoptotic cells are characterized by externalization of the phosphatidylserine (PS) phospholipid, which is normally located, in living cells, on the inner surface of the cell membrane. The phospholipid location on the outer membrane surface can be detected from early apoptosis up to complete cell degradation. Thus, use of the Annexin V recombinant protein, which shows a high affinity for phosphatidylserine conjugated to the Alexa Fluor 488 fluorescent dye, allows one to detect cell apoptosis with high accuracy. The use of Annexin V in combination with 7AAD allows simultaneous discrimination between viable cells (V–/7AAD–), early apoptotic cells (V+/7AAD–), and late apoptotic or necrotic cells (V+/7AAD+).

**Figure 3:**
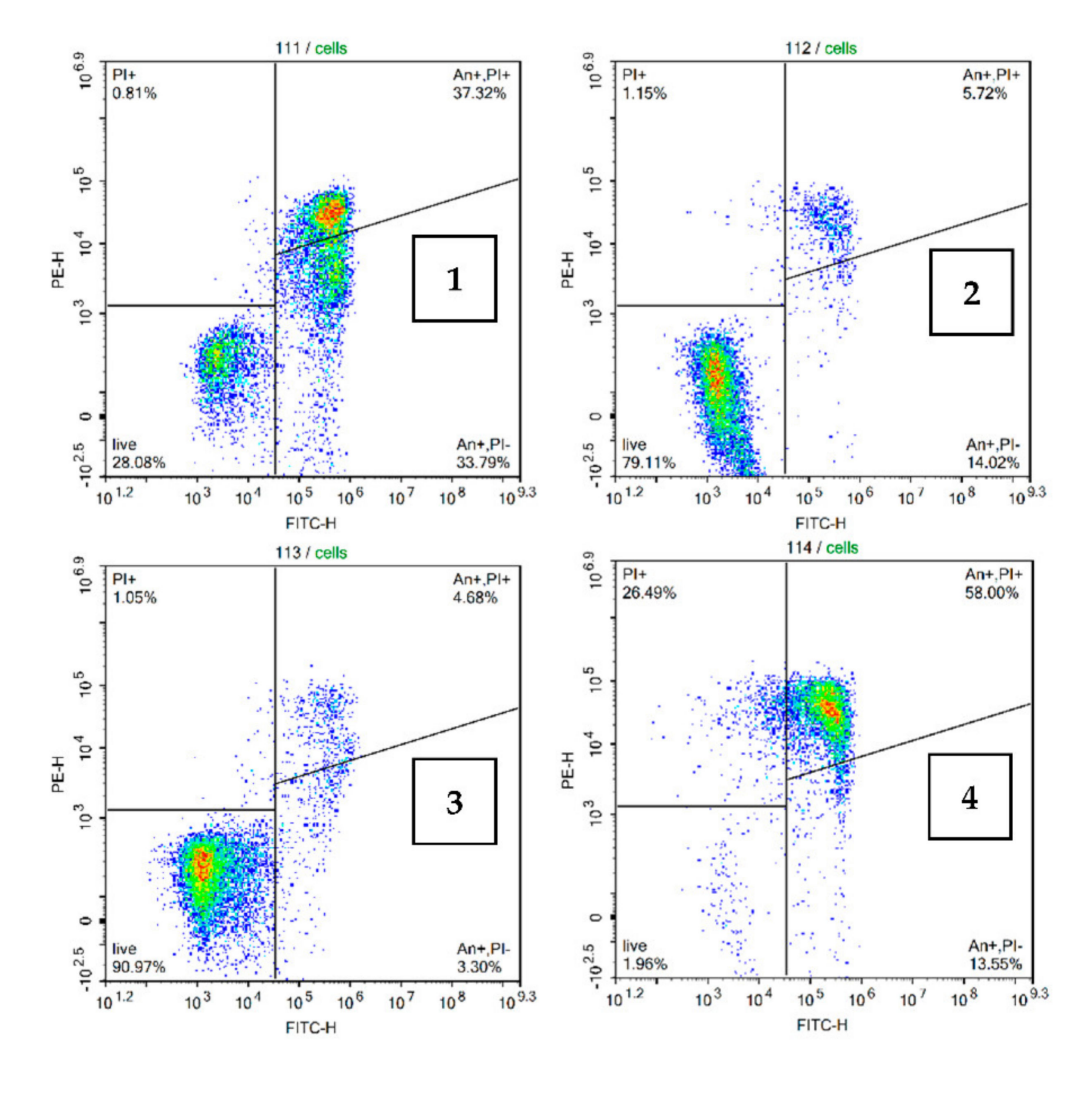

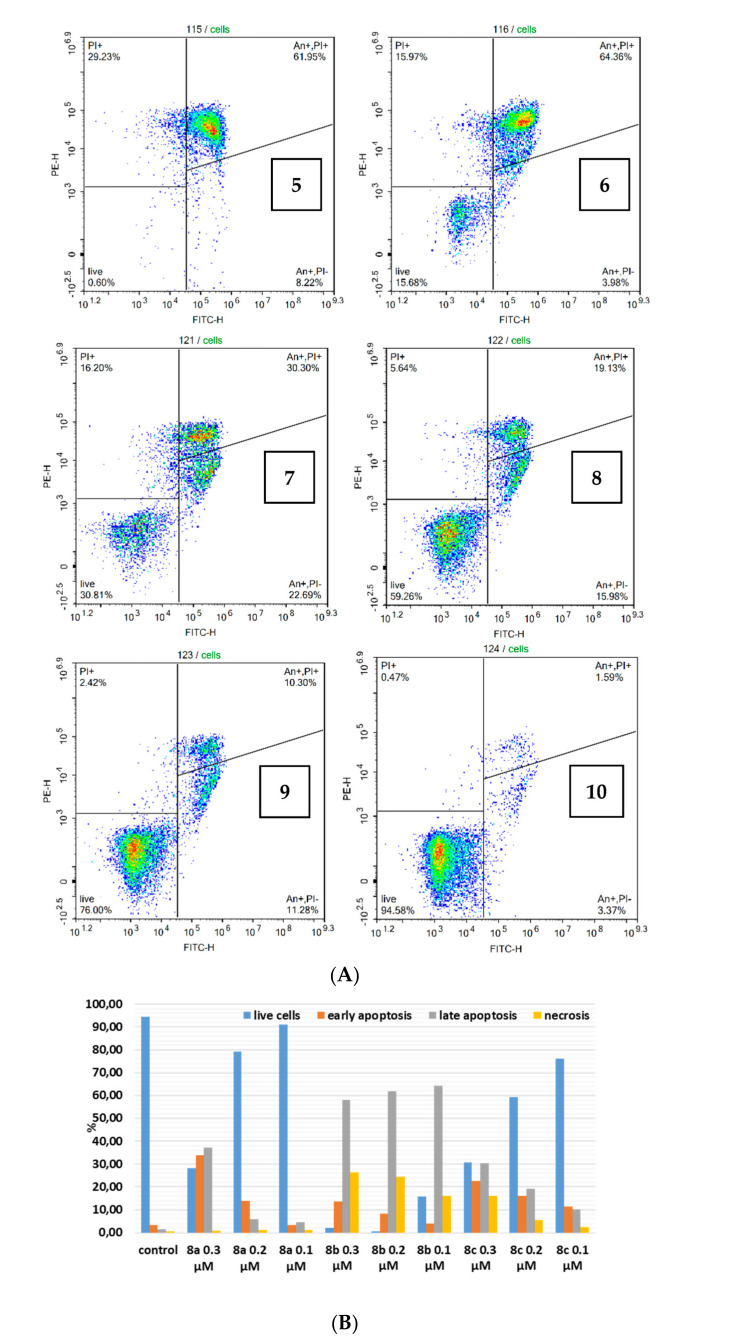
Cytofluorimetric analysis of the apoptosis-inducing activity of different macrocycles **8a**, **8b**, and **8c** in various concentrations on Jurkat cancer cells (**A**): (1–3, **8a**) 0.3 µM (1), 0.2 µM (2), 0.1 µM (3); (4–6, **8b**) 0.3 µM (4), 0.2 µM (5), 0.1 µM (6); (7–9, **8c**) 0.3 µM (7), 0.2 µM (8), 0.1 µM (9). Control (10); (**B**) Histogram of apoptosis phases in Jurkat cells treated with compounds **8a**, **8b,** and **8c**. The incubation time of macrocycles with the cells was 24 h.

The highest percentage of late apoptotic cells (64.36%) upon the addition of compound 8b to Jurkat cell culture was observed when the compound concentration was 0.1 µM (Figure 3, histogram 6), whereas at a concentration of 0.3 µM, necrotic cells predominated, with the percentage of late apoptosis being relatively high (26.49% and 58.0%, respectively). A somewhat different situation was found for compounds **8a** and **8c**. When present in the 0.3 µM concentration, compounds **8a** and **8c** showed similar patterns of predominance of early and late apoptosis in the equilibrium. In the cells treated with **8c**, a higher percentage of necrotic cells was observed (Figure 3, histograms 1–3 and 7–9). The effect of compounds **8a**, **8b**, and **8c** was dose dependent. To gain a better understanding of the mechanism of the apoptotic effect, we analyzed the probabilities of mitochondrial damage in the cells and the effect on the cell cycle.

**Figure 4:**
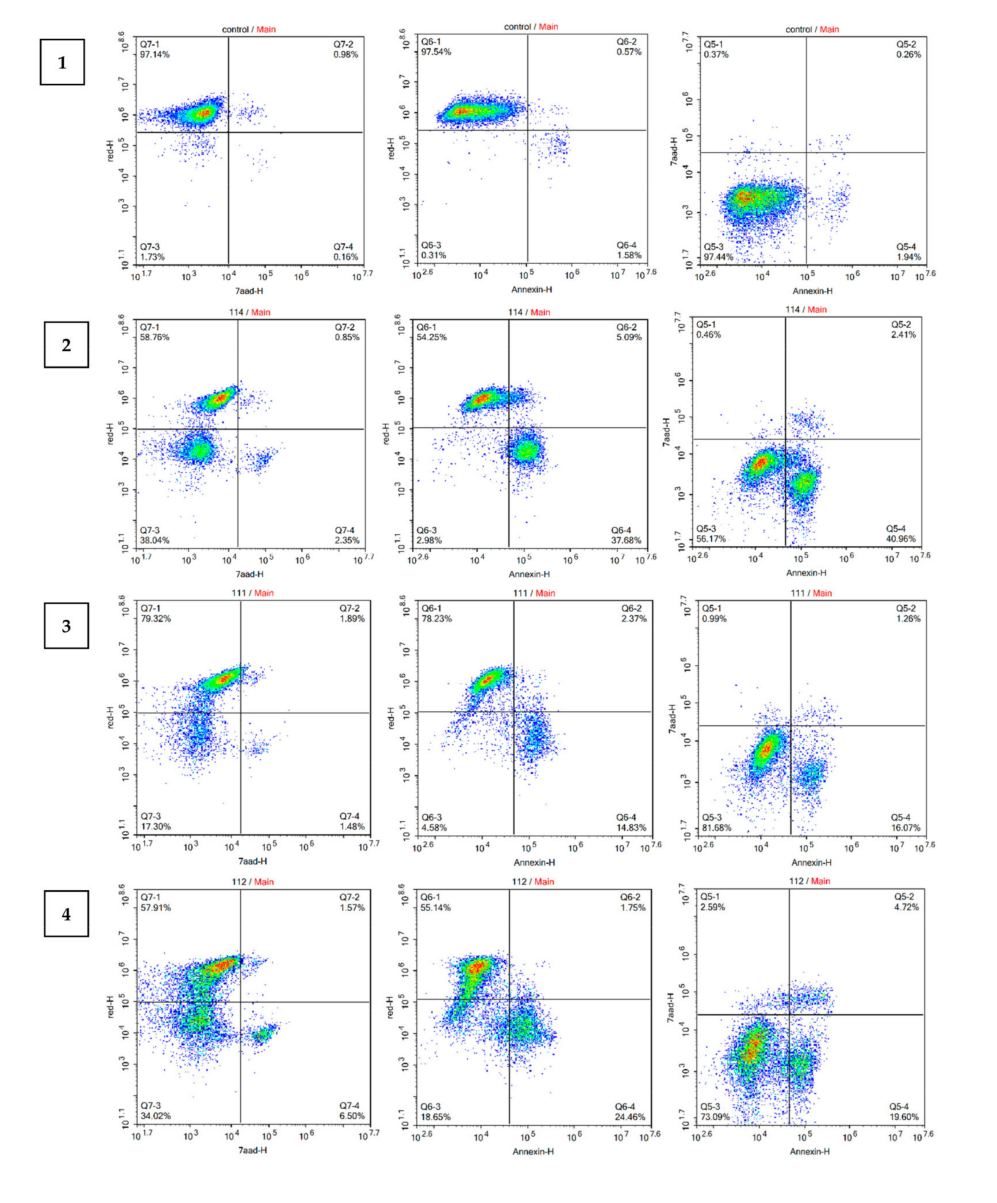

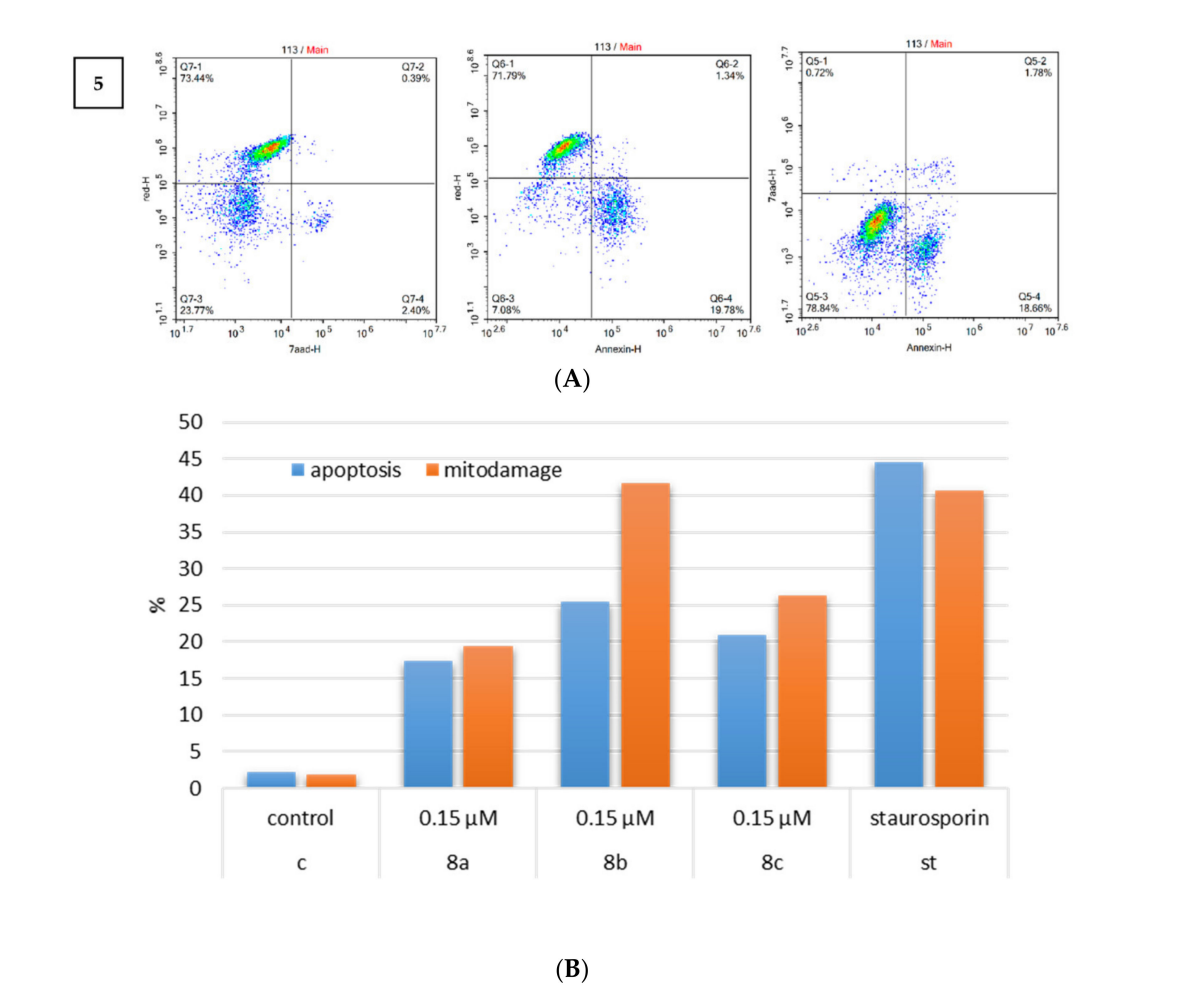
Detection of changes in the mitochondrial membrane potential (ΔΨ) and the coupled early and late apoptosis in Jurkat cells treated with compounds **8a**, **8b**, and **8c**. (**A**) (1), Control sample containing living cells; (2), staurosporine (0.2 µM); (3), **8a** (0.15 µM); (4), **8b** (0.15 µM); (5), **8c** (0.15 µM). The cells were stained with MitoSense Red, Annexin V–CF488A, and 7-AAD. The incubation time was 4 h. (**B**) Histogram of mitochondrial damage and apoptosis in Jurkat cells treated with compounds **8a**, **8b**, and **8c**.

The changes in the mitochondrial membrane potential (ΔΨ) in Jurkat cells induced by treatment with compounds **8a**, **8b**, and **8c** were detected using the MitoSense Red, a fluorescent dye, which is mainly accumulated in mitochondria and responds to changes in the membrane potential. The viable whole-body cells induce a high fluorescence level of MitoSense Red, whereas cells with dissipated mitochondrial membrane potential cause much lower fluorescence of MitoSense Red or no fluorescence. Annexin V and 7AAD are two dyes commonly used to detect apoptotic cells. The control cells show no fluorescence, whereas apoptotic cells demonstrate positive green fluorescence due to externalization of phosphatidylserine. 7-Aminoactinomycin (7-AAD) does not penetrate into living cells, but controls change in the permeability of cell membranes in late apoptosis and in dead cells. Thus, using three different dyes, MitoSense Red, Annexin V (CF488A), and 7-AAD, we analyzed the dissipation of the mitochondrial membrane potential and the related apoptosis in Jurkat tumor cells in response to the action of synthesized macrodiolides (Figure 4).

The study of the level of dissipation of the mitochondrial membrane potential (Δψ) revealed a statistically significant increase in the percentage of Jurkat tumor cells with reduced Δψ in the samples treated with compounds 8a (19.36%), 8b (41.71%), and 8c (26.26%). (Figure 4, histogram groups D, E, F). The most pronounced reduction of the mitochondrial potential was observed for compound 8a, this effect being dose dependent and comparable with the action of staurosporine, a known inhibitor of most protein kinases in the cells (Figure 4, histogram groups 3–5). While comparing the effect of the test compounds, one can see that the action of compound 8b is comparable with the action of staurosporine on mitochondria. The percentage of apoptotic cells in the samples treated with 8b (0.15 µM) is 58.15% (early apoptosis and late apoptosis) (Figure 4). These results indicate that apoptosis is initiated via the internal (mitochondrial) pathway, with compound 8b inducing the most pronounced dissipation of the mitochondrial potential because of marked suppression of the oxidation and phosphorylation in the mitochondria of Jurkat cells.

#### 2.2.3. Cell Cycle Analysis

**Figure 5:**
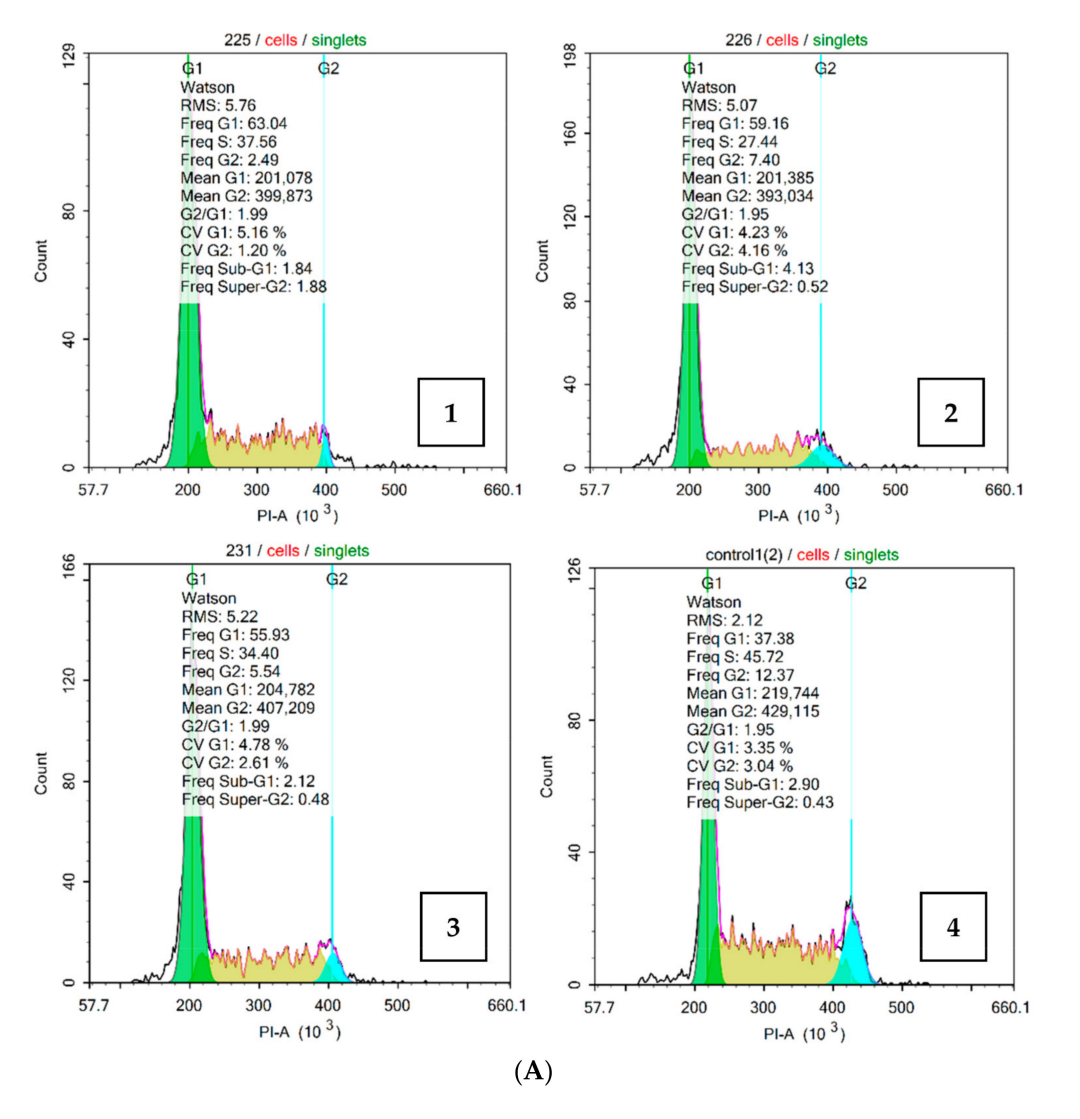

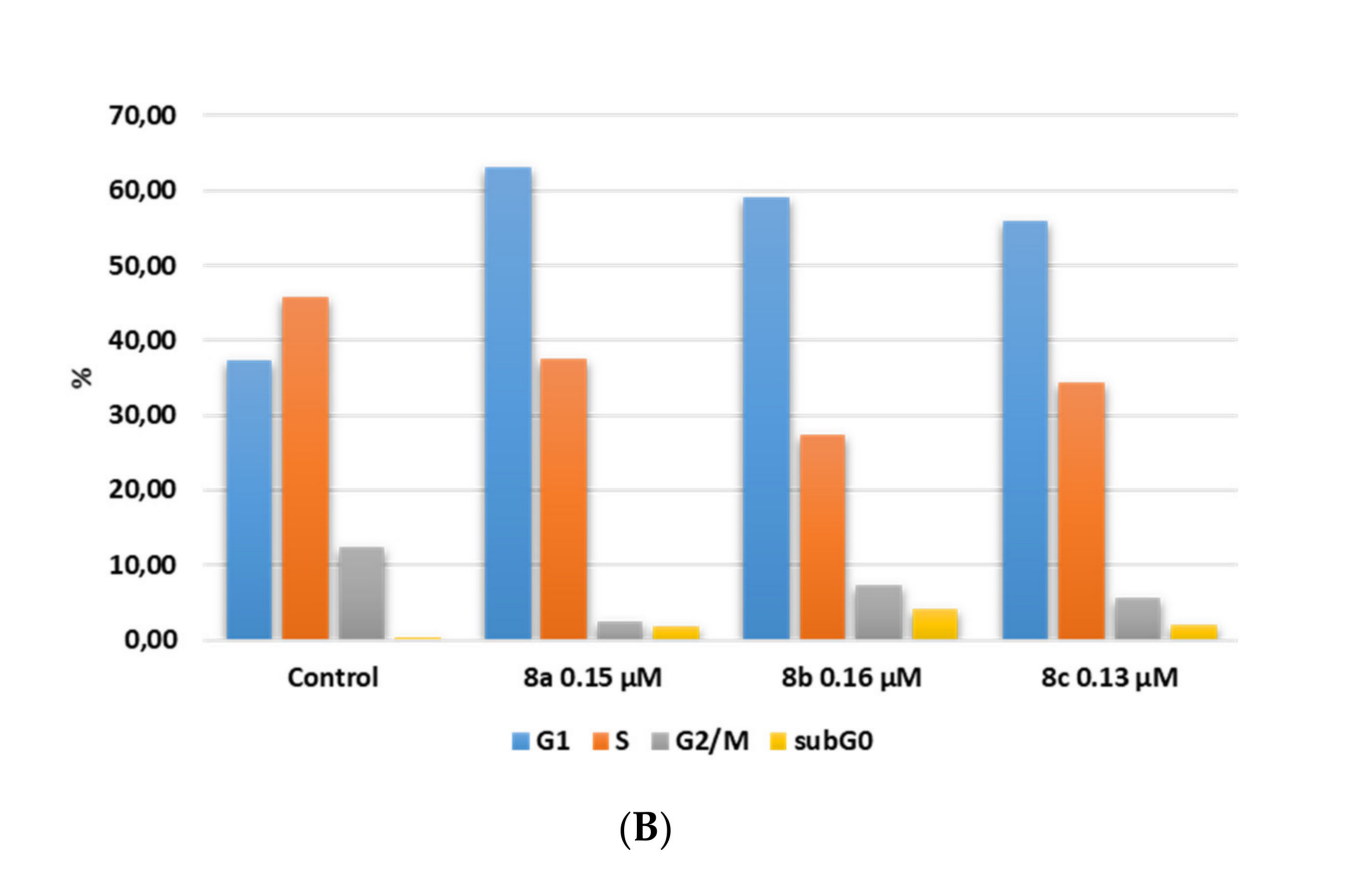
Cell cycle phases for Jurkat cells treated with macrocycles **8a**, **8b**, and **8c** at IC50 concentrations. (**A**) (1) 0.15 µM (**8a**); (2) 0.16 µM (**8b**); (3) 0.13 µM (**8c**). Control (4); The incubation time of macrocycles with the cells was 24 h. (**B**) Histogram of cell cycle phasis in Jurkat cells treated with compounds **8a, 8b,** and **8c.**

Analysis of the cell cycle in the control sample showed a balance of cell division processes, typical of most tumor cultures, particularly a predominance of cells in S and G1/G0 phases. Indeed, the percentage of cells in G0 phase was 37.38%, that in S phase was 45.72%, while 12.37% of cells were in G2/M phase. The cell population in the subG0 phase was 0.43%. Twenty-four hours after the exposure of Jurkat cells to compounds **8a**, **8b**, and **8c**, first, processes of apoptosis predominated (increase in the sub-G0-G1 range; Figure 5, histograms 1,2,3); second, the percentage of cells in G0/G1 phase increased for all three samples up to 63.04%, 59.16%, and 55.93%, respectively; and third, the percentage of S-phase cells somewhat decreased in comparison with the control (Figure 5, histograms 1–4). 

The greatest number of cells in the sub-G0-G1 range was found for the samples treated with compound **8b** in a concentration of 0.16 µM (4.13%) (histogram 2). Furthermore, the percentage of G2/M phase cells decreased in all samples. Thus, the above results may be indicative of a cytotoxic activity of compounds **8a**, **8b**, and **8c** against chronic myeloid and T cell leukemia cells, caused by the ability of this group of compounds to induce apoptosis. Additionally, all of the compounds that we synthesized here would, most likely, have immunosuppressive activity caused by their anti-mitogenic effect.

## 3. Materials and Methods

### 3.1. Chemistry

^1^H, ^13^C NMR spectra were obtained using a Bruker AVANCE 400 in CDCl_3_ operating at 400.13 MHz for ^1^H, 100.62 MHz for ^13^C and Bruker Ascend-500 (500 MHz (^1^H), 125 MHz (^13^C). High-resolution mass spectra of compounds were recorded on a Bruker maXis spectrometer (tandem quadrupole/time-of-flight mass analyzer) equipped with an electrospray ionization source (ESI) and matrix-activated laser desorption/ionization (MALDI). UV spectra were acquired on a Shimadzu UV-1800 UV/Vis spectrometer (l = 1 cm) in CH_2_Cl_2_. The individuality and purity of the synthesized compounds were controlled using of TLC on Silufol UV-254 plates; anisic aldehyde in acetic acid was used as a developer. All solvents were dried (diethyl ether, toluene over Na, dioxane over NaOH, methanol over Mg, chloroform, dichloromethane over P_2_O_5_) and freshly distilled before use. All reactions were carried out under a dry argon atmosphere. Commercially available alkynols (Aldrich, St. Louis, MO, USA), Cp_2_TiCl_2_ (Aldrich) were used. α,ω-Alca-n*Z*,(n+4)*Z*-dienols were synthesized according to procedures described in the literature [17].

### 3.2. Chemical Experimental Data

#### General Procedure Synthesis of Cyclophanes

To a mixture of α,ω-alka-n*Z*,(n+4)*Z*-dienediol (0.2 mmol, 1 eq), aromatic dicarboxylic acid (0.2 mmol, 1 eq) and 4-dimethylaminopyridine (12 mg, 0.1 mmol, 0.5 eq) in dichloromethane (35 mL) at 0 °C was added a solution of EDC·HCl (62 mg, 0.4 mmol, 2 eq) in 5 mL of dichloromethane under argon. The mixture was warmed up to room temperature and stirred for 12 h. After, the reaction mixture was treated with a 5% solution of HCl in H_2_O (2 × 10 mL). The products were extracted with dichloromethane (2 × 30 mL), the extracts were dried with MgSO_4_, the solvent was evaporated, and the residue was chromatographed on a column (SiO_2_, elution with petroleum ether/EtOAc (10/1)).

(10Z,14Z)-7,8,9,12,13,16,17,18,26,31-decahydro-5H,20H-tribenzo[c,g,k][1,5,10,14]tetraoxacyclohexacosine-5,20-dione (**6a**)

White waxy solid; yield 48%. R_f_ = 0.52, hexane/EtOAc 3:1. UV(CH_2_Cl_2_) λ_max_(lgε) 294 nm (3.84). ^1^H NMR (400 MHz, CDCl_3_): δ = 7.85 (d, *J* = 7.8 Hz, 2H), 7.52–7.43 (m, 6H), 7.04 (t, *J* = 7.8 Hz, 4H), 5.33 (d, *J* = 5.9 Hz, 4H), 5.18 (s, 4H), 4.37–4.27 (m, 4H), 2.09 (dd, *J* = 13.2, 6.4 Hz, 4H), 2.01 (s, 4H), 1.81–1.70 (m, 4H); ^13^C NMR (101 MHz, CDCl_3_): δ = 167.2, 157.7, 136.3, 133.2, 131.9, 130.0, 128.9, 127.3, 121.2, 120.6, 113.3, 70.2, 64.6, 28.6, 27.1, 23.7. ESI-MS: calcd. for C_34_H_36_O_6_ + Na^+^ [M + Na]^+^ 563.2410; found 563.2408.

(13Z,17Z)-2,6,9,22-tetraoxa-1,7(1,2),4(1,3)-tribenzenacyclotricosaphane-13,17-diene-8,23-dione (**6b**)

White waxy solid; yield 53%. R_f_ = 0.54, hexane/EtOAc 3:1. UV(CH_2_Cl_2_) λ_max_(lgε) 297 nm (3.89).^1^H NMR (400 MHz, CDCl_3_): δ = 7.83 (d, *J* = 7.3 Hz, 2H), 7.54 (s, 1H), 7.49–7.37 (m, 5H), 7.03 (t, *J* = 8.1 Hz, 4H), 5.36–5.23 (m, 4H), 5.17 (s, 4H), 4.31 (t, *J* = 6.1 Hz, 4H), 2.05 (dd, *J* = 13.7, 6.8 Hz, 4H), 2.00–1.88 (m, 4H), 1.71 (dt, *J* = 13.7, 6.8 Hz, 4H); ^13^C NMR (101 MHz, CDCl_3_): δ = 167.4, 157.7, 137.0, 133.2, 131.9, 130.2, 128.8, 128.7, 126.9, 125.9, 121.5, 120.7, 113.6, 70.6, 64.4, 28.6, 27.1, 23.5. ESI-MS: calcd. for C_34_H_36_O_6_ + H^+^ [M + H]^+^ 541.2590; found 541.2585.

(13Z,17Z)-2,6,9,22-tetraoxa-1,7(1,2),4(1,4)-tribenzenacyclotricosaphane-13,17-diene-8,23-dione (**6c**)

White waxy solid; yield 57%. R_f_ = 0.52, hexane/EtOAc 5:1. UV(CH_2_Cl_2_) λ_max_(lgε) 295 nm (3.86). ^1^H NMR (400 MHz, CDCl_3_): δ = 7.87–7.82 (m, 2H), 7.65 (dd, *J* = 5.4, 3.4 Hz, 2H), 7.49–7.35 (m, 4H), 7.03 (t, *J* = 7.6 Hz, 4H), 5.40–5.26 (m, 8H), 4.24 (t, *J* = 6.1 Hz, 4H), 2.04 (q, *J* = 7.1 Hz, 4H), 1.99–1.92 (m, 4H), 1.71–1.62 (m, 4H); ^13^C NMR (101 MHz, CDCl_3_): δ = 166.9, 157.7, 134.5, 133.3, 131.9, 130.0, 128.8, 128.4, 128.3, 121.2, 120.7, 113.5, 68.8, 64.4, 28.5, 27.2, 23.5. ESI-MS: calcd. for C_34_H_36_O_6_ + Na^+^ [M + Na]^+^ 563.2410; found 563.2404.

(11Z,15Z)-7,8,9,10,13,14,17,18,19,20,28,33-dodecahydro-5H,22H-tribenzo[c,g,k][1,5,10,14]tetraoxacyclooctacosine-5,22-dione (**7a**)

White waxy solid.; yield 54%. R_f_ = 0.56, hexane/EtOAc 3:1. UV(CH_2_Cl_2_) λ_max_(lgε) 296 nm (3.84). ^1^H NMR (400 MHz, CDCl_3_): δ = 7.83 (dd, *J* = 7.8, 1.5 Hz, 2H), 7.51 (s, 4H), 7.44 (dd, *J* = 11.5, 4.2 Hz, 2H), 7.02 (dd, *J* = 7.8, 5.4 Hz, 4H), 5.41–5.30 (m, 4H), 5.19 (s, 4H), 4.34 (t, *J* = 6.6 Hz, 4H), 2.04 (dd, *J* = 11.7, 4.9 Hz, 8H), 1.79–1.70 (m, 4H), 1.51–1.41 (m, 4H); ^13^C NMR (101 MHz, CDCl_3_): δ = 167.1, 157.7, 136.4, 133.1, 131.8, 129.7, 129.6, 127.3, 121.5, 120.7, 113.7, 70.4, 64.9, 28.1, 27.3, 26.6, 25.9. ESI-MS: calcd. for C_36_H_40_O_6_ + Na^+^ [M + Na]^+^ 591.2723; found 591.2717

(14Z,18Z)-2,6,9,24-tetraoxa-1,7(1,2),4(1,3)-tribenzenacyclopentacosaphane-14,18-diene-8,25-dione (**7b**)

White waxy solid; yield 62%. R_f_ = 0.54, hexane/EtOAc 3:1. UV(CH_2_Cl_2_) λ_max_(lgε) 293 nm (3.87). ^1^H NMR (500 MHz, CDCl_3_): δ =7.86–7.80 (m, 2H), 7.52–7.42 (m, 6H), 7.04 (dd, *J* = 12.5, 5.8 Hz, 4H), 5.38–5.28 (m, 4H), 5.19 (d, *J* = 9.8 Hz, 4H), 4.37–4.29 (m, 4H), 2.11–1.90 (m, 8H), 1.76–1.65 (m, 4H), 1.49–1.38 (m, 4H); ^13^C NMR (126 MHz, CDCl_3_): δ = 167.4, 157.7, 137.0, 133.1, 131.8, 129.7, 129.6, 128.8, 126.8, 125.8, 121.6, 120.7, 113.7, 70.6, 65.1, 28.2, 27.3, 26.7, 25.9. ESI-MS: calcd. for C_36_H_40_O_6_ + H^+^ [M + H]^+^ 569.2903; found 569.2898

(14Z,18Z)-2,6,9,24-tetraoxa-1,7(1,2),4(1,4)-tribenzenacyclopentacosaphane-14,18-diene-8,25-dione (**7c**)

White waxy solid; yield 65%. R_f_ = 0.54, hexane/EtOAc 3:1. UV(CH_2_Cl_2_) λ_max_(lgε) 297 nm (3.84).^1^H NMR (400 MHz, CDCl_3_): δ = 7.85 (dd, *J* = 7.8, 1.5 Hz, 2H), 7.67 (dd, *J* = 5.4, 3.4 Hz, 2H), 7.49–7.37 (m, 4H), 7.08–7.00 (m, 4H), 5.41–5.24 (m, 8H), 4.26 (t, *J* = 6.8 Hz, 4H), 2.10–1.92 (m, 8H), 1.70–1.54 (m, 4H), 1.36 (dd, *J* = 14.9, 7.6 Hz, 4H); ^13^C NMR (101 MHz, CDCl_3_): δ = 167.1, 157.6, 134.6, 133.3, 132.0, 129.7, 128.5, 128.4, 128.3, 121.2, 120.7, 113.3, 68.6, 65.2, 28.1, 27.4, 26.6, 25.9. ESI-MS: calcd. for C_36_H_40_O_6_ + Na^+^ [M + Na]^+^ 591.2723; found 591.2714

(13*Z*,17*Z*)-7,8,9,10,11,12,15,16,19,20,21,22,23,24,32,37-hexadecahydro-5*H*,26*H*-tribenzo[c,g,k][1,5,10,14]tetraoxacyclodotriacontine-5,26-dione (**8a**)

White waxy solid; yield 57%. R_f_ = 0.58, hexane/EtOAc 3:1. UV(CH_2_Cl_2_) λ_max_(lgε) 296 nm (3.78). ^1^H NMR (400 MHz, CDCl_3_): δ = 7.83 (d, *J* = 7.8 Hz, 2H), 7.52 (s, 4H), 7.44 (dd, *J* = 11.0, 4.6 Hz, 2H), 7.02 (t, *J* = 7.6 Hz, 4H), 5.41–5.30 (m, 4H), 5.19 (s, 4H), 4.33 (t, *J* = 6.6 Hz, 4H), 2.12–1.96 (m, 8H), 1.73 (dd, *J* = 13.2, 5.9 Hz, 4H), 1.46–1.30 (m, 12H); ^13^C NMR (101 MHz, CDCl_3_): δ = 166.9, 157.8, 136.4, 133.1, 131.7, 130.2, 129.3, 127.2, 121.4, 120.6, 113.7, 70.3, 65.1, 29.4, 28.6, 27.4, 26.9, 25.7. ESI-MS: calcd. for C_40_H_48_O_6_ + H^+^ [M + H]^+^ 625.3529; found 625.3524.

(16Z,20Z)-2,6,9,28-tetraoxa-1,7(1,2),4(1,3)-tribenzenacyclononacosaphane-16,20-diene-8,29-dione (**8b**)

White waxy solid; yield 63%. R_f_ = 0.54, hexane/EtOAc 3:1. UV(CH_2_Cl_2_) λ_max_(lgε) 295 nm (3.82).^1^H NMR (500 MHz, CDCl_3_): δ = 7.83 (dd, *J* = 8.0, 1.8 Hz, 2H), 7.55 (s, 1H), 7.52–7.34 (m, 4H), 7.06–7.00 (m, 4H), 5.44–5.30 (m, 4H), 5.19 (s, 4H), 4.31 (t, *J* = 6.7 Hz, 4H), 2.09–1.96 (m, 8H), 1.74–1.67 (m, 5H), 1.40–1.28 (m, 12H); ^13^C NMR (126 MHz, CDCl_3_): δ = 167.1, 157.8, 137.0, 133.1, 131.7, 130.1, 129.3, 128.8, 126.8, 125.9, 121.6, 120.7, 113.8, 70.6, 65.1, 29.4, 28.7, 28.6, 27.5, 26.9, 25.8. ESI-MS: calcd. for C_40_H_48_O_6_ + Na^+^ [M + Na]^+^ 647.3349; found 647.3343

(16Z,20Z)-2,6,9,28-tetraoxa-1,7(1,2),4(1,4)-tribenzenacyclononacosaphane-16,20-diene-8,29-dione (**8c**)

White waxy solid; yield 71%. R_f_ = 0.60, hexane/EtOAc 3:1. UV(CH_2_Cl_2_) λ_max_(lgε) 296 nm (3.83).^1^H NMR (400 MHz, CDCl_3_): δ = 7.85 (dd, *J* = 7.8, 1.0 Hz, 2H), 7.66 (dd, *J* = 5.1, 3.7 Hz, 2H), 7.42 (ddd, *J* = 9.8, 8.3, 5.4 Hz, 4H), 7.10–6.96 (m, 4H), 5.47–5.29 (m, 8H), 4.24 (t, *J* = 6.8 Hz, 4H), 2.15–1.97 (m, 8H), 1.67–1.56 (m, 4H), 1.28 (d, *J* = 9.3 Hz, 12H); ^13^C NMR (101 MHz, CDCl_3_): δ = 166.9, 157.7, 134.6, 133.3, 131.9, 130.2, 129.3, 128.5, 128.3, 121.1, 120.6, 113.3, 68.6, 65.2, 29.4, 28.8, 28.6, 27.6, 27.0, 25.8. ESI-MS: calcd. for C_40_H_48_O_6_ + NH_4_^+^ [M + NH_4_]^+^ 642.3795; found 642.3789.

### 3.3. Biology Studies

#### 3.3.1. Cell Culturing

Cells (Jurkat, K562, U937, HL-60, HEK293, and normal fibroblasts) were purchased from HPA Culture Collections (Salisbury, UK) and cultured according to standard mammalian tissue culture protocols and the sterile technique. All cell lines used in the study were tested and shown to be free of mycoplasma and other viral contamination. HEK293 and fibroblast cell lines were cultured as monolayers and maintained in Dulbecco’s modified Eagle’s medium (DMEM, Gibco BRL, Waltham, MA, USA) supplemented with 10% fetal bovine serum and 1% penicillin-streptomycin solution at 37 °C in a humidified incubator under a 5% CO_2_ atmosphere.

Cells were maintained in RPMI 1640 (Jurkat, K562, U937, HL60) (Gibco, Waltham, MA, USA) supplemented with 4 mM glutamine, 10% FBS (Sigma, Burlington, MA, USA), and 100 units/ml penicillin–streptomycin (Sigma, Burlington, MA, USA). All types of cells were grown in an atmosphere of 5% CO2 at 37 °C. The cells were subcultured at 2–3-day intervals. Adherent cells (HEK293 and normal fibroblasts) were suspended using trypsin/EDTA and counted after they reached 80% confluency. Cells were then seeded in 24-well plates at 5 × 10^4^ cells per well and incubated overnight. Jurkat, K562, U937, and HL60 cells were subcultured at 2-day intervals with a seeding density of 1 × 10^5^ cells per 24-well plates in RPMI with 10% FBS.

#### 3.3.2. Cytotoxicity Assay

Viability (live/dead) assessment was performed by staining cells with 7-AAD (7-Aminoactinomycin D) (Biolegend, Fell, Germany). After treatment, cells were harvested, washed 1–2 times with phosphate-buffered saline (PBS), and centrifuged at 400× *g* for 5 min. Cell pellets were resuspended in 200 mL of flow cytometry staining buffer (PBS without Ca^2+^ and Mg^2+^, 2.5% FBS) and stained with 5 μL of 7-AAD staining solution for 15 min at room temperature in the dark. Samples were acquired on a NovoCyteTM 2000 FlowCytometry System (ACEA) equipped with a 488 nm argon laser. Detection of 7-AAD emission was collected through a 675/30 nm filter in FL4 channel.

#### 3.3.3. Viability and Apoptosis

Induction of apoptosis tests were carried out following the known procedure [17]. Apoptosis was determined by flow cytometric analysis of Annexin V and 7-aminoactinomycin D staining. After treatment, cells during 24 h were harvested, washed 1–2 times with phosphate-buffered saline (PBS), and centrifuged at 400× *g* for 5 min. Cell pellets were resuspended in 200 μL of flow cytometry staining buffer (PBS without Ca^2+^and Mg^2+^, 2.5% FBS). Then, 200 μL of Guava Nexin reagent (Millipore, Bedford, MA, USA) were added to 5 × 10^5^ cells in 200 μL, and the cells were incubated with the reagent for 20 min at room temperature in the dark. At the end of incubation, the cells were analyzed on a NovoCyteTM 2000 FlowCytometry System (ACEA, San Diego, CA, USA).

#### 3.3.4. Cell Cycle Analysis

Cell cycle analysis was carried out following the known procedure [17]. Guava Cell Cycle Reagent (Millipore, Burlington, MA, USA) was used. Samples were analyzed on a NovoCyteTM 2000 FlowCytometry System (ACEA, San Diego, CA, USA).

#### 3.3.5. Mitochondrial Damage

Mitochondrial damage tests were carried out following the known procedure [17]. Cytometric assay (Millipore’s FlowCellect™MitoDamage Kit, Merck, Burlington, MA, USA) that allowed multiparametric evaluation of three cell health markers: change in mitochondrial potential (early apoptosis and cellular stress), phosphatidylserine expression on the cell surface (late apoptosis), and membrane permeabilization (cell death), was used.

## 4. Conclusions

Thus, we developed an original method for stereoselective synthesis of previously unreported cyclophanes containing a 1Z,5Z-diene moiety in their structure with good yields and high stereoselectivity (more than 98%). The developed method includes synthesis of α,ω-alka-nZ,(n+4)Z-dienediols based on the use of original Ti-catalyzed intermolecular homo-cyclomagnesiation of O-containing 1,2-dienes with Grignard reagents followed by their intermolecular cyclocondensation with aromatic dicarboxylic acids mediated by EDC·HCl and DMAP. The studied biological activity revealed that the new cyclophanes were found to exhibit in vitro cytotoxic activity toward Jurkat, K562, U937, HL-60, and cancer cell lines. The mechanism of apoptotic cell death upon the interaction with the macrodiolides was identified. The induction of apoptosis by these compounds is due to dissipation of the mitochondrial membrane potential of the tumor cell. The most promising candidate is compound **8b**, the effect of which is comparable with the effect of staurosporine. Cell cycle analysis provided data on the effect of new macrodiolides on all cell cycle phases, especially G2/M, which may also be indicative of an antitumor action of this group of compounds. 

## Figures and Tables

**Table 1:** Cytotoxic activities in vitro of synthesized cyclophanes **6**‒**8** measured on cell cultures (Jurkat, K562, U937, HL-60, Hek293, and normal fibroblasts) (µM).

Comp.	Jurkat (IC_50_, µM) *	K562 (IC_50_, µM) *	U937 (IC_50_, µM) *	HL-60 (IC_50_, µM) *	Hek293 (IC_50_, µM) *	Fibrobl. (IC_50_, µM) *
**6a**	0.39 ± 0.03	0.61 ± 0.05	0.49 ± 0.04	0.43 ± 0.03	1.94 ± 0.17	4.71 ± 0.42
**6b**	0.48 ± 0.05	0.64 ± 0.06	0.51 ± 0.05	0.44 ± 0.04	2.18 ± 0.21	5.09 ± 0.47
**6c**	0.34 ± 0.03	0.58 ± 0.05	0.46 ± 0.04	0.40 ± 0.03	1.76 ± 0.16	4.22 ± 0.39
**7a**	0.49 ± 0.04	0.76 ± 0.08	0.56 ± 0.06	0.94 ± 0.08	3.86 ± 0.34	7.12 ± 0.59
**7b**	0.94 ± 0.08	1.49 ± 0.11	1.17 ± 0.09	0.97 ± 0.09	4.21 ± 0.39	7.69 ± 0.72
**7c**	0.35 ± 0.03	0.59 ± 0.04	0.39 ± 0.03	0.89 ± 0.09	3.47 ± 0.31	6.55 ± 0.61
**8a**	0.15 ± 0.02	0.37 ± 0.02	0.30 ± 0.02	0.22 ± 0.02	1.44 ± 0.11	3.37 ± 0.29
**8b**	0.16 ± 0.01	0.39 ± 0.04	0.31 ± 0.03	0.24 ± 0.02	1.68 ± 0.14	3.48 ± 0.32
**8c**	0.13 ± 0.01	0.34 ± 0.03	0.27 ± 0.02	0.19 ± 0.01	1.21 ± 0.09	3.19 ± 0.28

* Data are presented as the mean ± SEM calculated from results of at least 3 independent experiments.

## Data Availability

The data presented in this study are available in this article.

## Supplementary Materials

The following are available online at https://www.mdpi.com/article/10.3390/ijms22168787/s1.
