# Supplementary material for: Direct Synthesis of Polyaromatic Cyclophanes Containing Bis-Methylene-Interrupted Z-Double Bonds and Study of Their Antitumor Activity In Vitro
Source: Int J Mol Sci. 2021 Aug 16;22(16):8787. doi: 10.3390/ijms22168787 (PMC8396040; doi:10.3390/ijms22168787)
Supplement: Supplementary file 1 [file ijms-22-08787-s001.zip › ijms-1324648-supplementary.pdf]

Direct synthesis of polyaromatic cyclophanes containing bis-methylene-interrupted *Z*-double bonds and studying their antitumor activity *in vitro*

Vladimir A. D'yakonov,*[a] Ilgiz I. Islamov,[a] LilyaU. Dzhemileva,*[a] Elina Kh. Makarova[a], and Usein M. Dzhemilev[a]

[a] *Institute of Petrochemistry and Catalysis, Russian Academy of Sciences, 141 Prospekt Oktyabrya, Ufa 450075, Russian Federation; e-mail: DyakonovVA@gmail.com*

Table of Contents

^{1}H NMR and ^{13}C NMR spectra of compounds 6a-8c……………………......3

^{1}H NMR and ^{13}C NMR spectra of compounds 6a-8c

6a

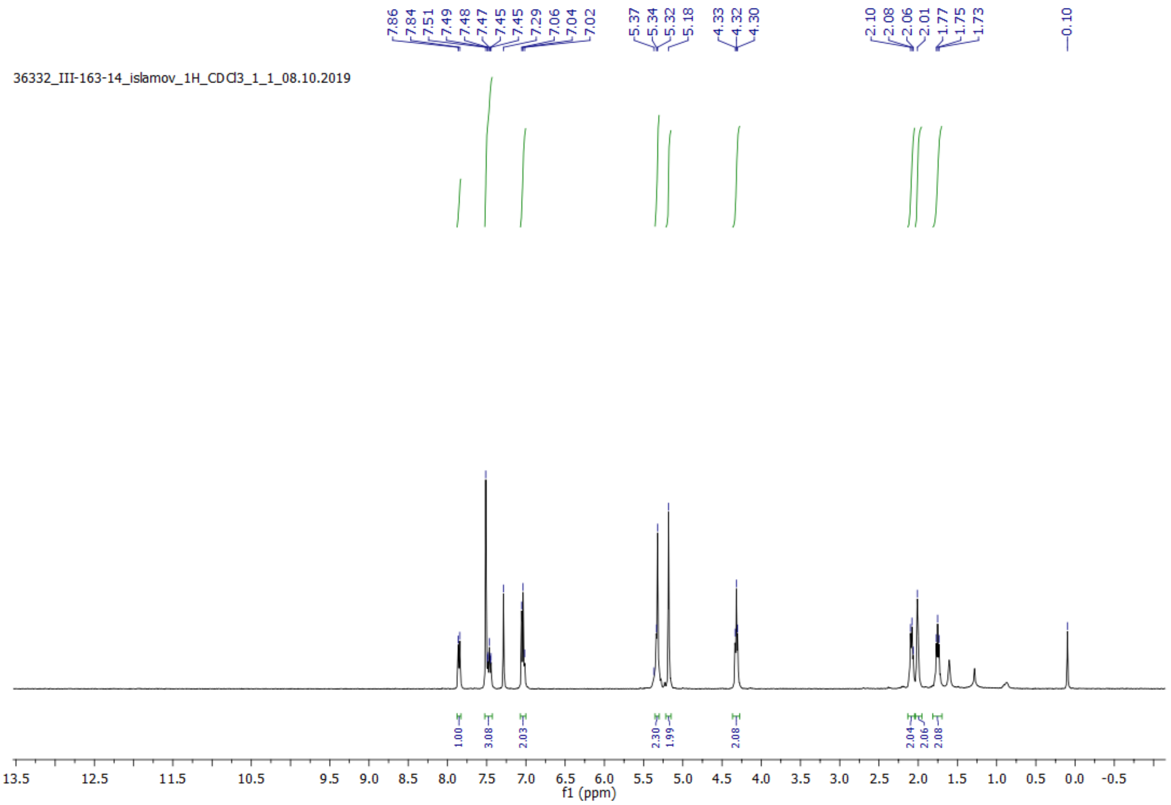

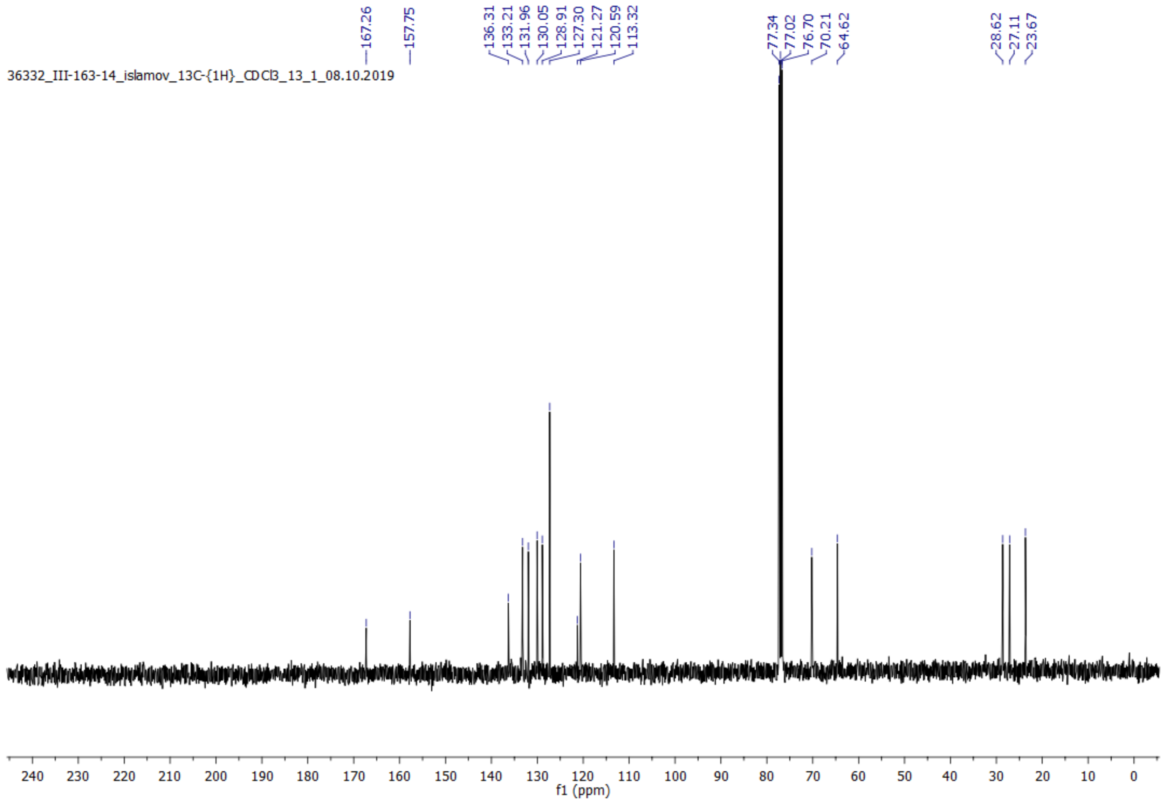

6b

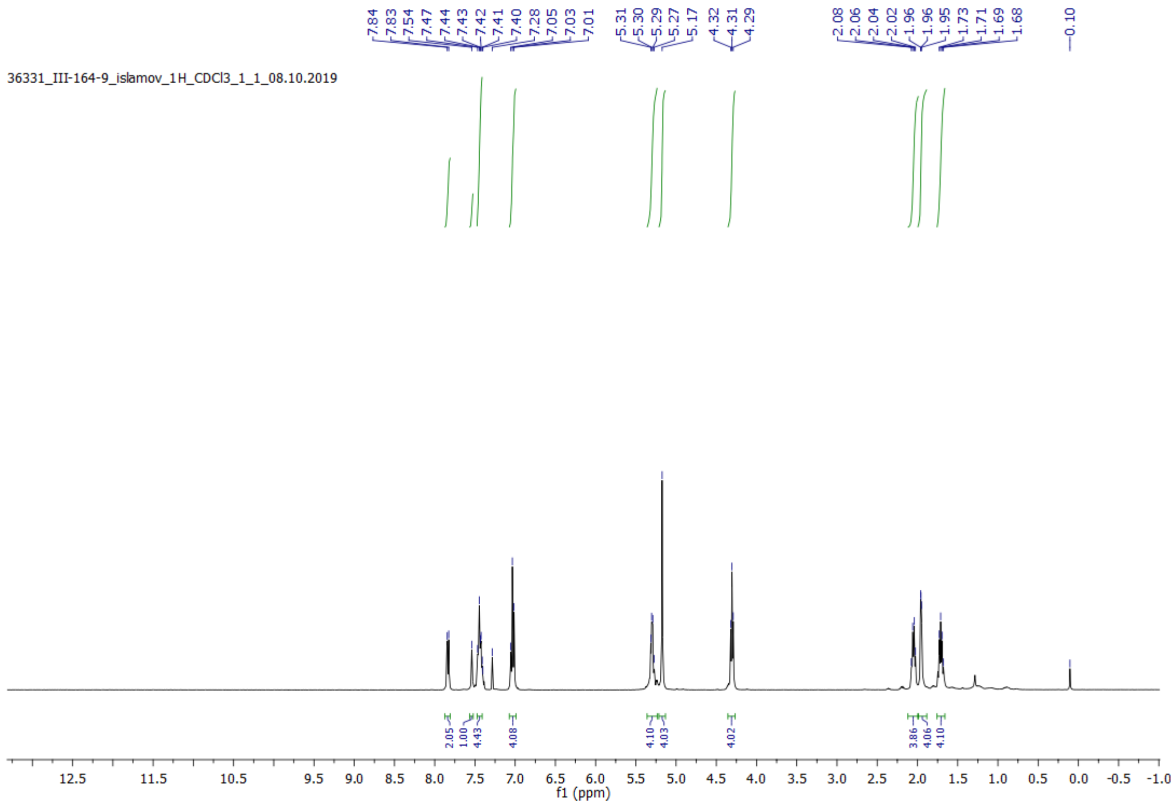
36331_III-164-9_islamov_1H_CDCl3_1_1_08.10.2019
7.84 7.83 7.54 7.47 7.44 7.43 7.42 7.41 7.40 7.28 7.05 7.03 7.01
5.31 5.30 5.29 5.27 5.17 4.32 4.31 4.29
2.08 2.06 2.04 2.02 1.96 1.95 1.73 1.71 1.69 1.68
0.10
2.05 1.00 4.43 4.08 4.10 4.03 4.02 3.86 4.06 4.10
f1 (ppm)

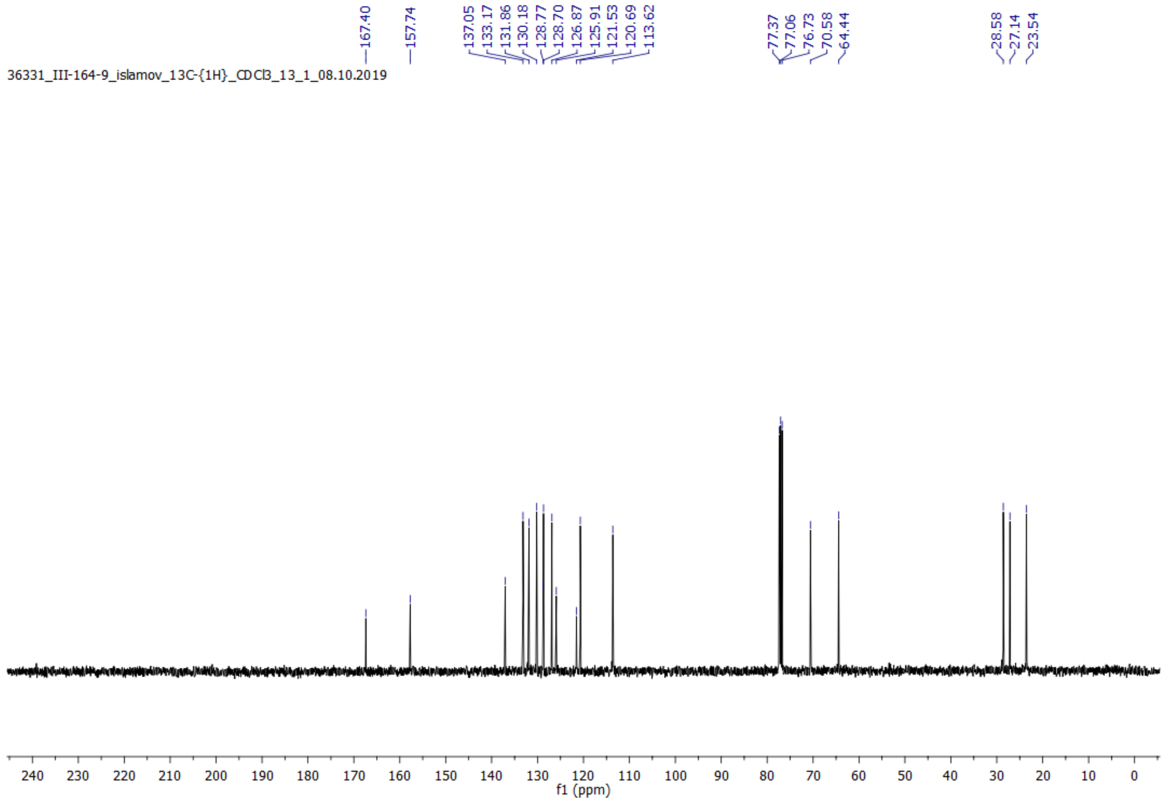
36331_III-164-9_islamov_13C-{1H}_CDCl3_13_1_08.10.2019
167.40 157.74
137.05 133.17 131.86 130.18 128.77 128.70 126.87 125.91 121.53 120.69 113.62
77.37 77.06 76.73 70.58 64.44
28.58 27.14 23.54
f1 (ppm)

6c

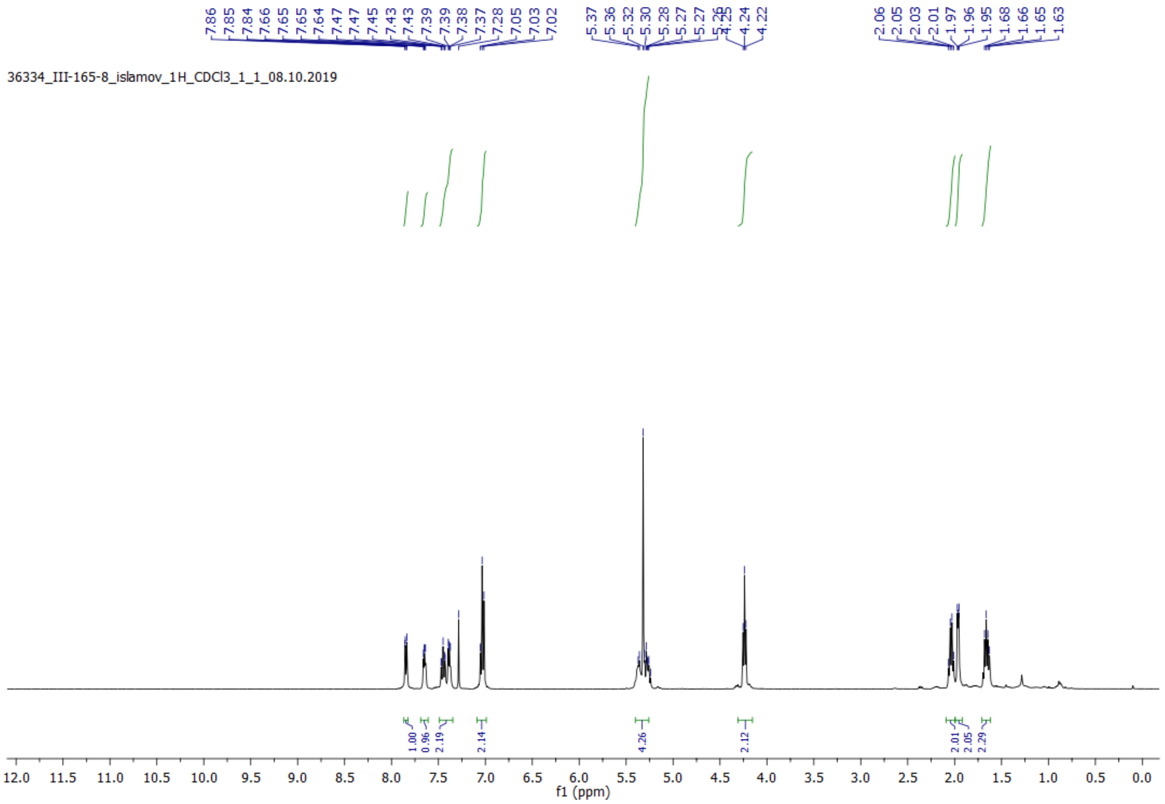
36334_III-165-8_islamov_1H_CDCl3_1_1_08.10.2019
f1 (ppm)

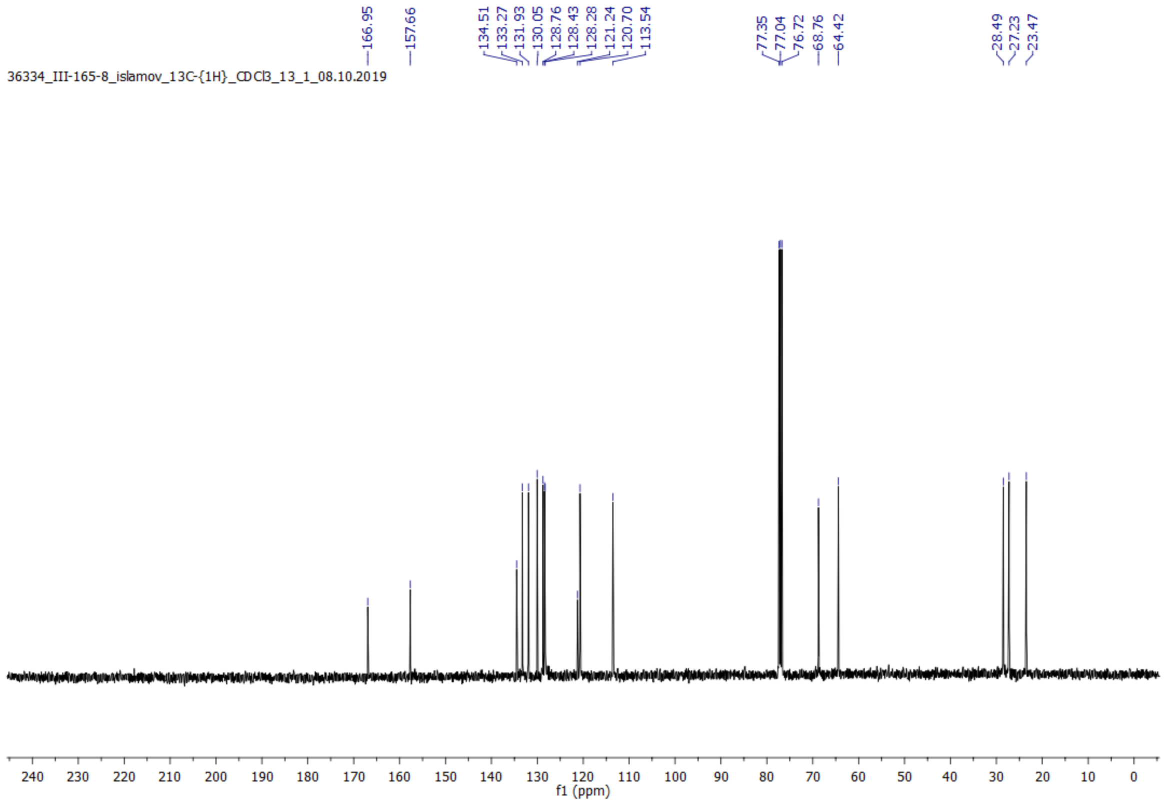
36334_III-165-8_islamov_13C-{1H}_CDCl3_13_1_08.10.2019
f1 (ppm)

7a

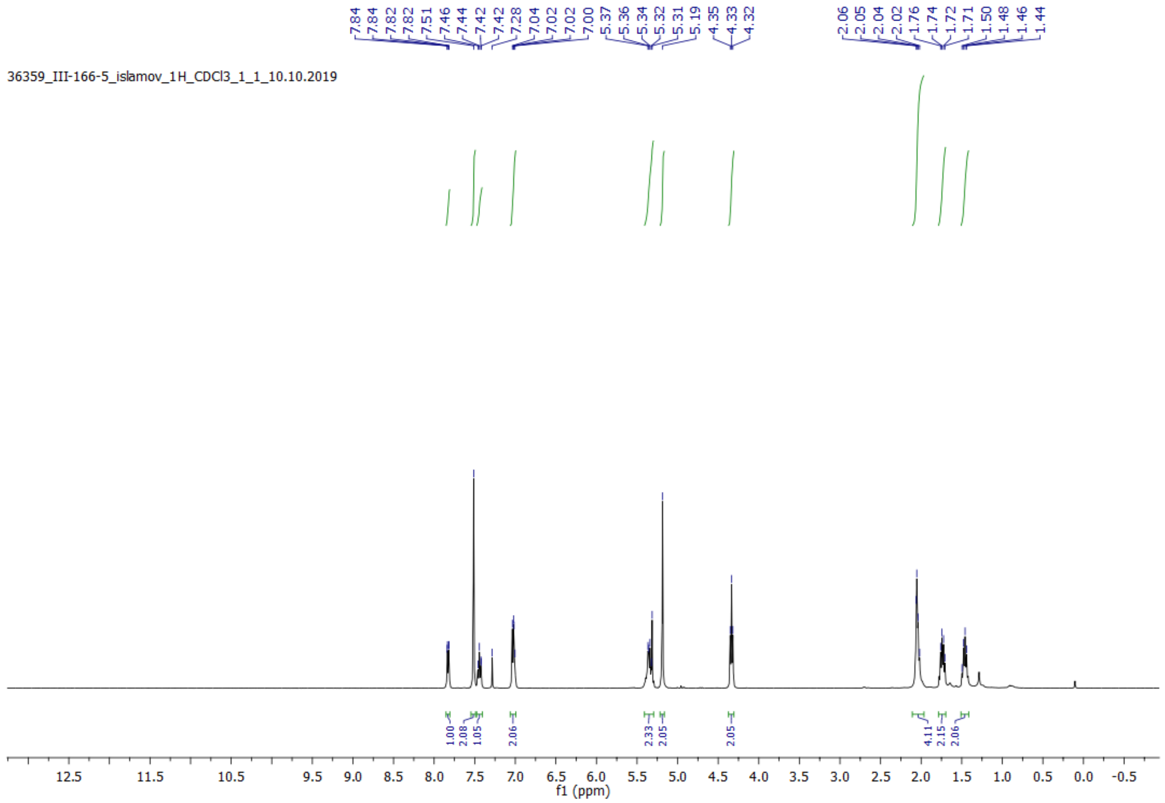
36359_III-166-5_islamov_1H_CDCl3_1_1_10.10.2019
f1 (ppm)

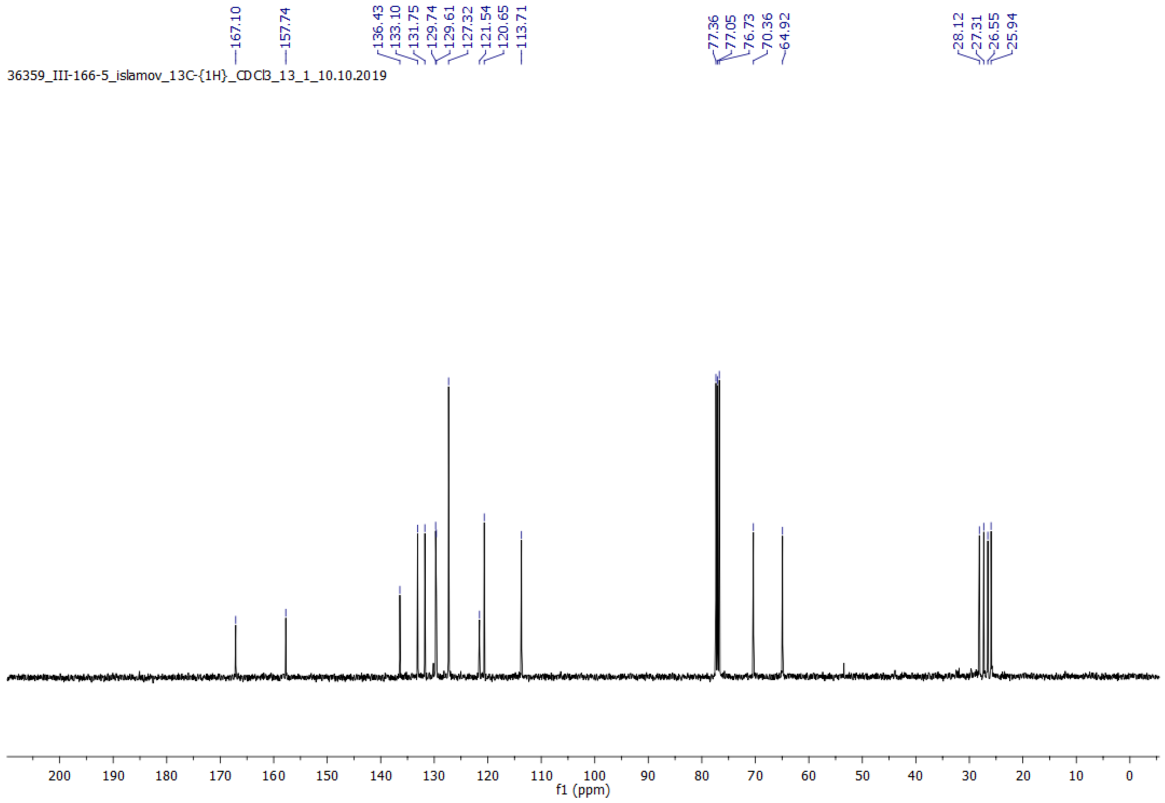
36359_III-166-5_islamov_13C-{1H}_CDCl3_13_1_10.10.2019
f1 (ppm)

7b

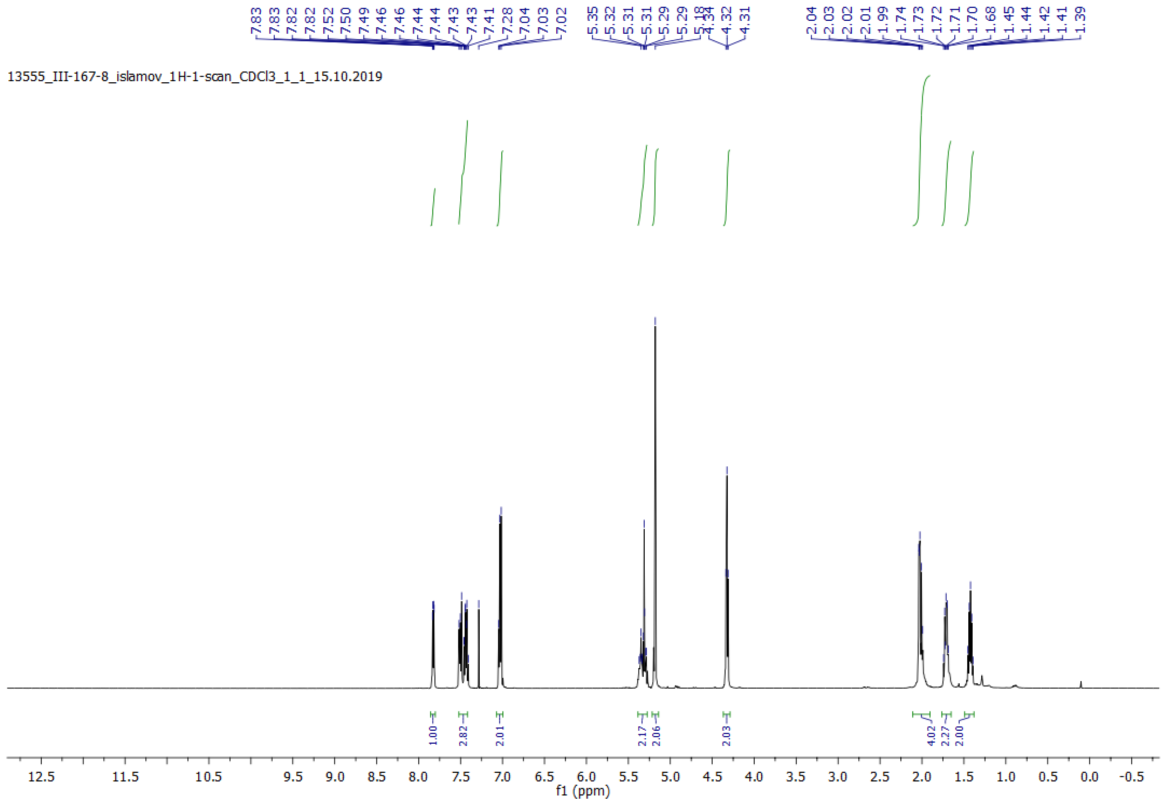
13555_III-167-8_islamov_1H-1-scan_CDCl3_1_1_15.10.2019
f1 (ppm)

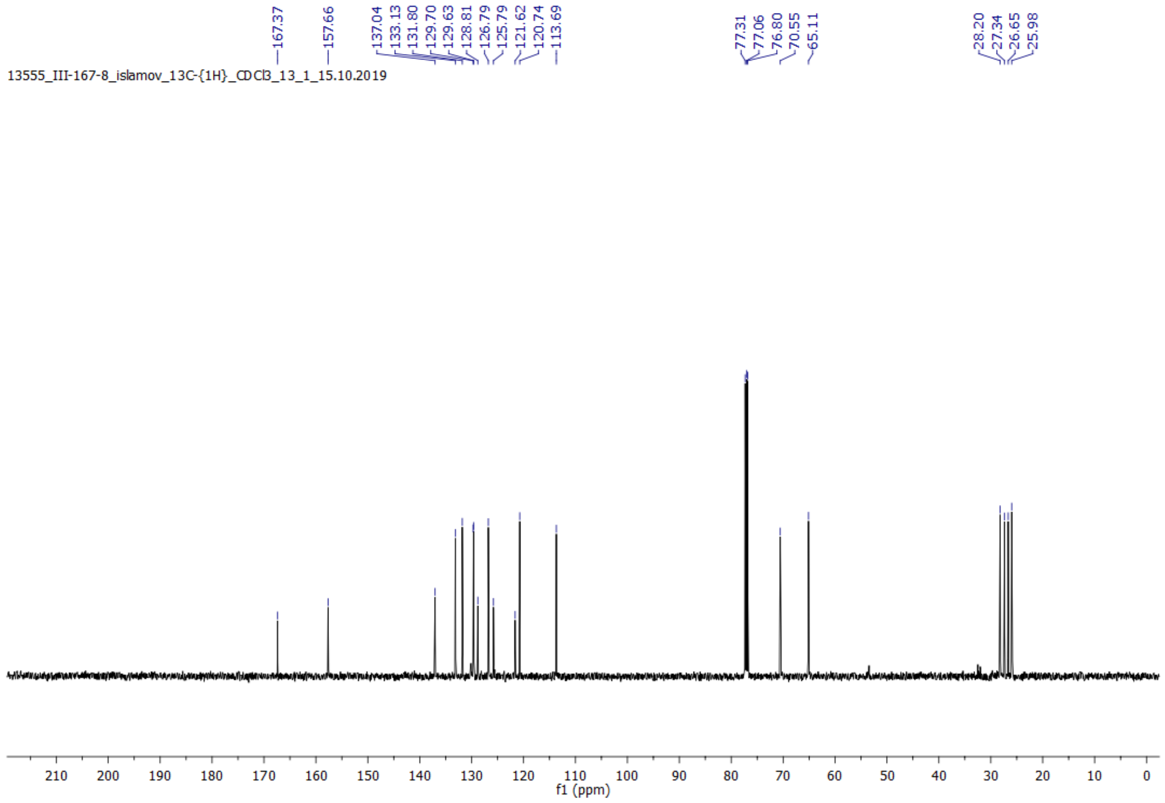
13555_III-167-8_islamov_13C-{1H}_CDCl3_13_1_15.10.2019
f1 (ppm)

7c

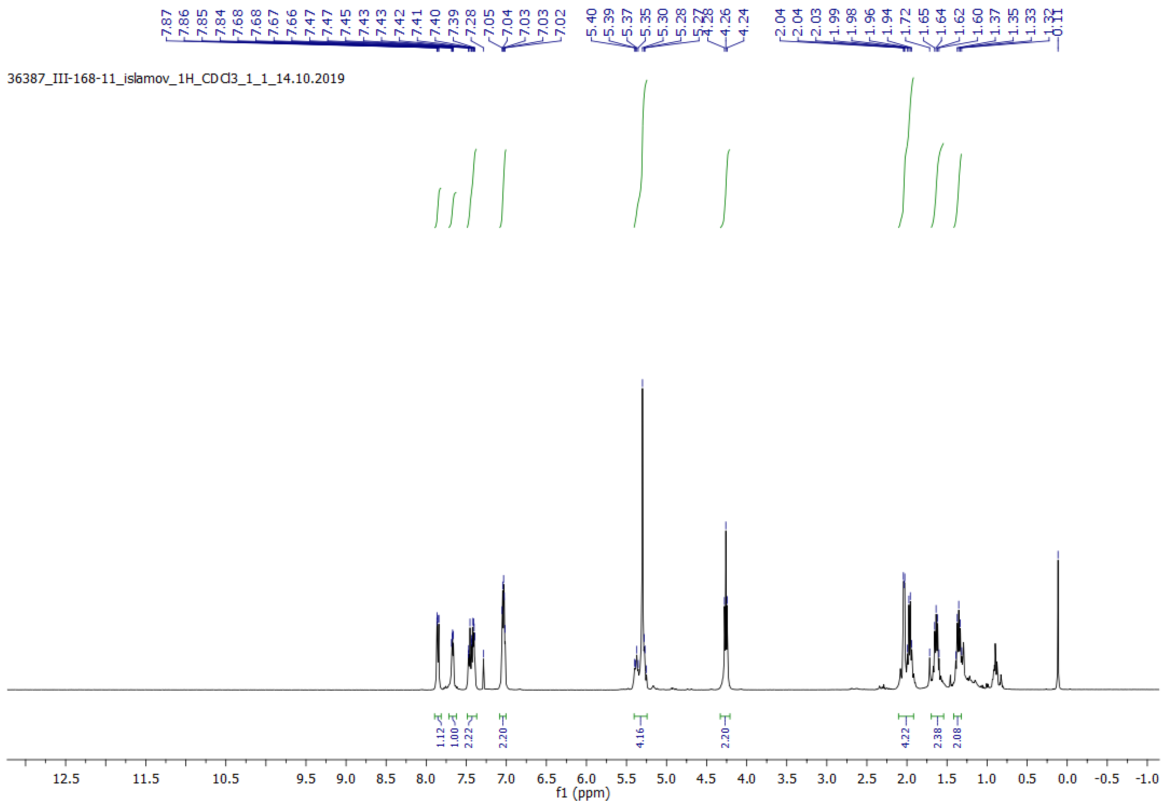

36387_III-168-11_islamov_1H_CDCl3_1_1_14.10.2019
f1 (ppm)

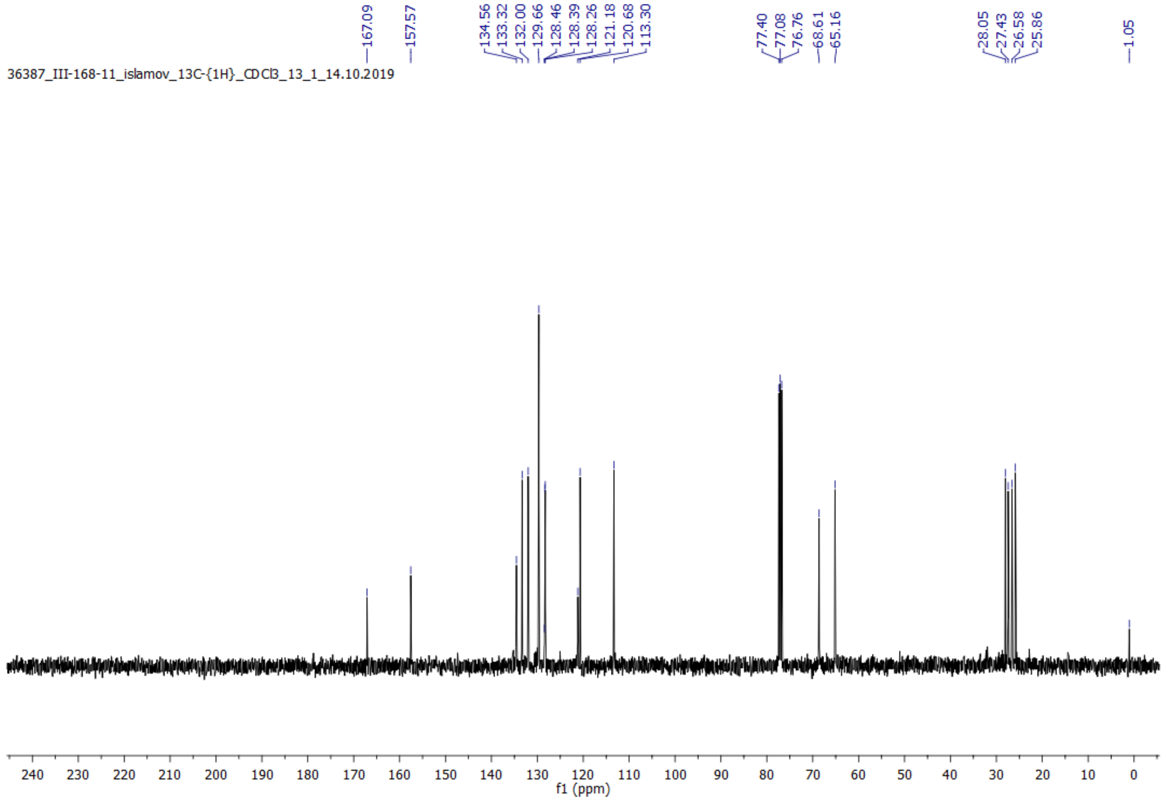

36387_III-168-11_islamov_13C-{1H}_CDCl3_13_1_14.10.2019
f1 (ppm)

8a

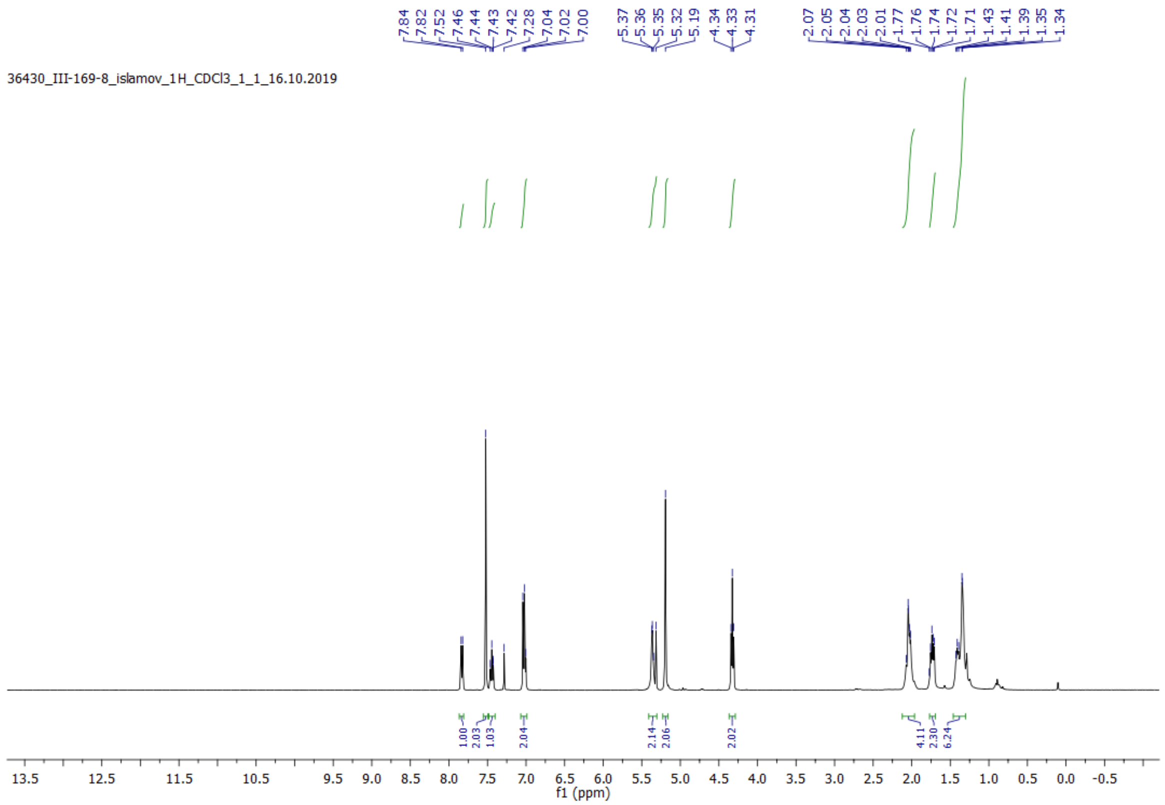
36430_III-169-8_islamov_1H_CDCl3_1_1_16.10.2019
f1 (ppm)

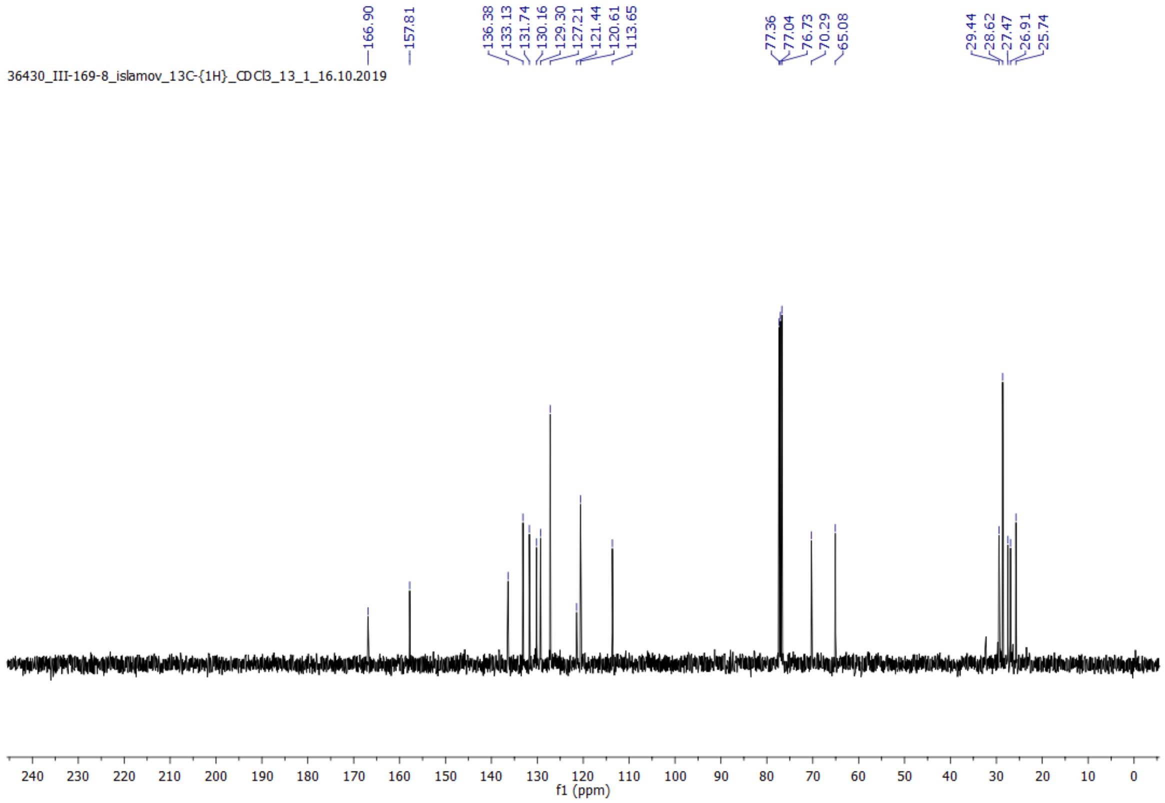
36430_III-169-8_islamov_13C-{1H}_CDCl3_13_1_16.10.2019
f1 (ppm)

8b

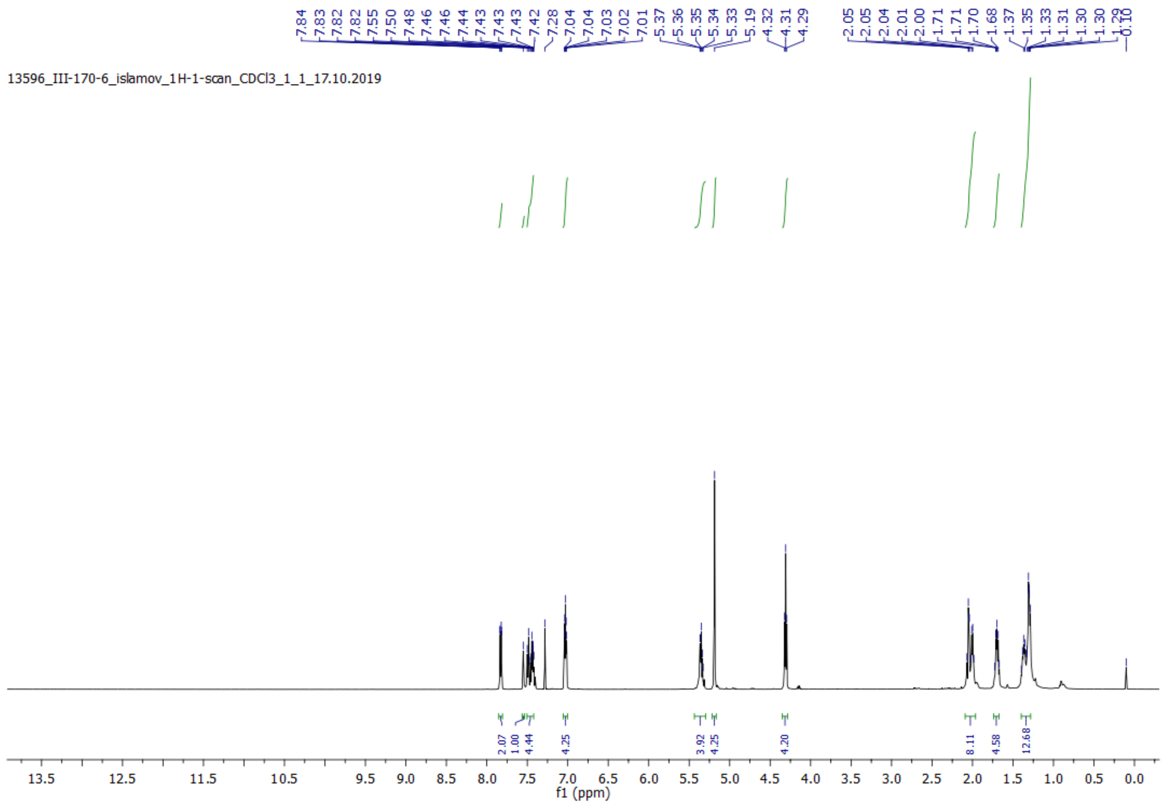
13596_III-170-6_islamov_1H-1-scan_CDCl3_1_1_17.10.2019
7.84 7.83 7.82 7.82 7.55 7.50 7.48 7.46 7.46 7.44 7.43 7.43 7.42 7.28 7.04 7.04 7.03 7.02 7.01 5.37 5.36 5.35 5.34 5.33 5.19 4.32 4.31 4.29 2.05 2.05 2.04 2.01 2.00 1.71 1.71 1.70 1.68 1.37 1.35 1.33 1.31 1.30 1.30 1.29 0.10
2.07 1.00 4.44 4.25 3.92 4.25 4.20 8.11 4.58 12.68
f1 (ppm)

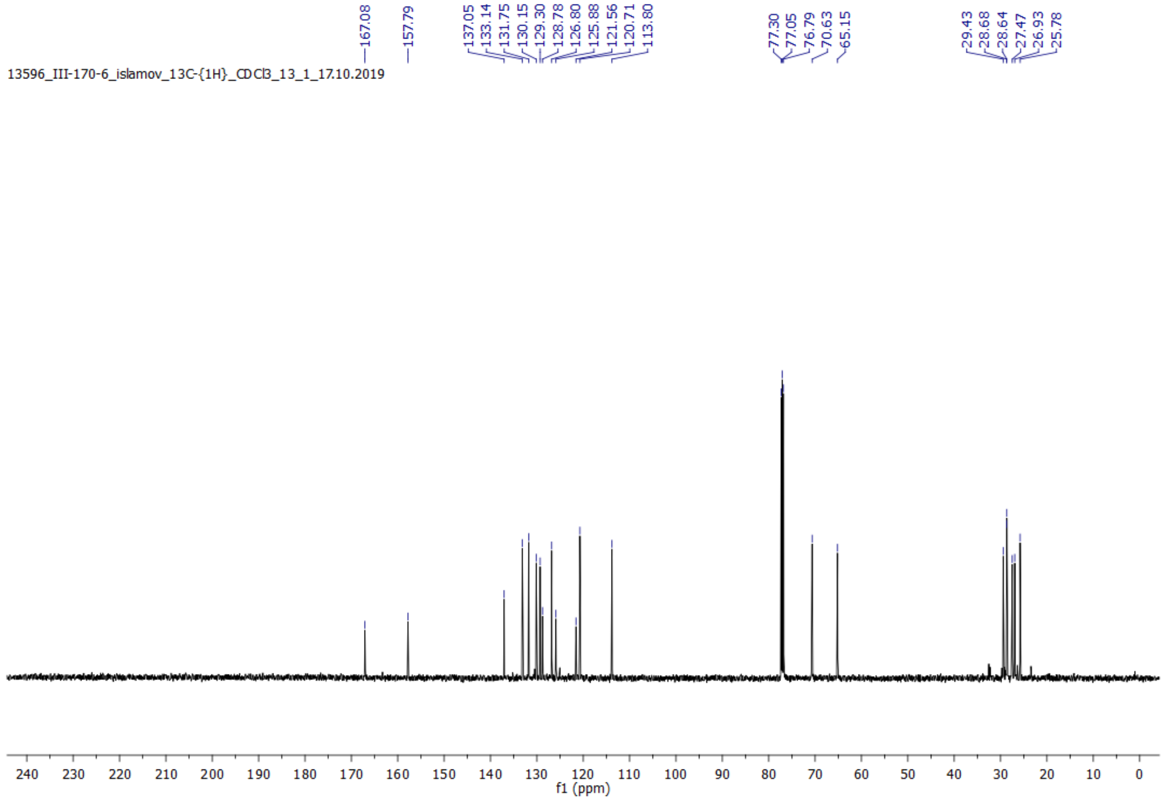
13596_III-170-6_islamov_13C-{1H}_CDCl3_13_1_17.10.2019
167.08 157.79 137.05 133.14 131.75 130.15 129.30 128.78 126.80 125.88 121.56 120.71 113.80 77.30 77.05 76.79 70.63 65.15 29.43 28.68 28.64 27.47 26.93 25.78
f1 (ppm)

8c

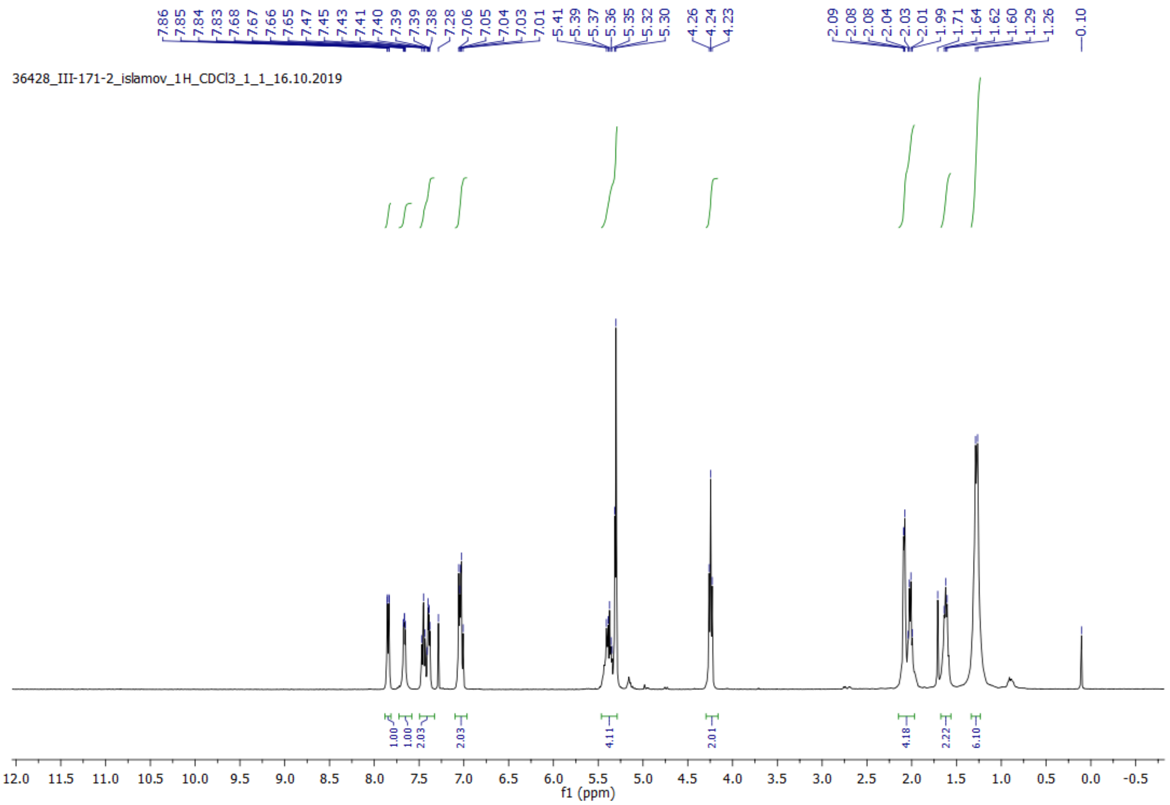
36428_III-171-2_islamov_1H_CDCl3_1_1_16.10.2019
f1 (ppm)

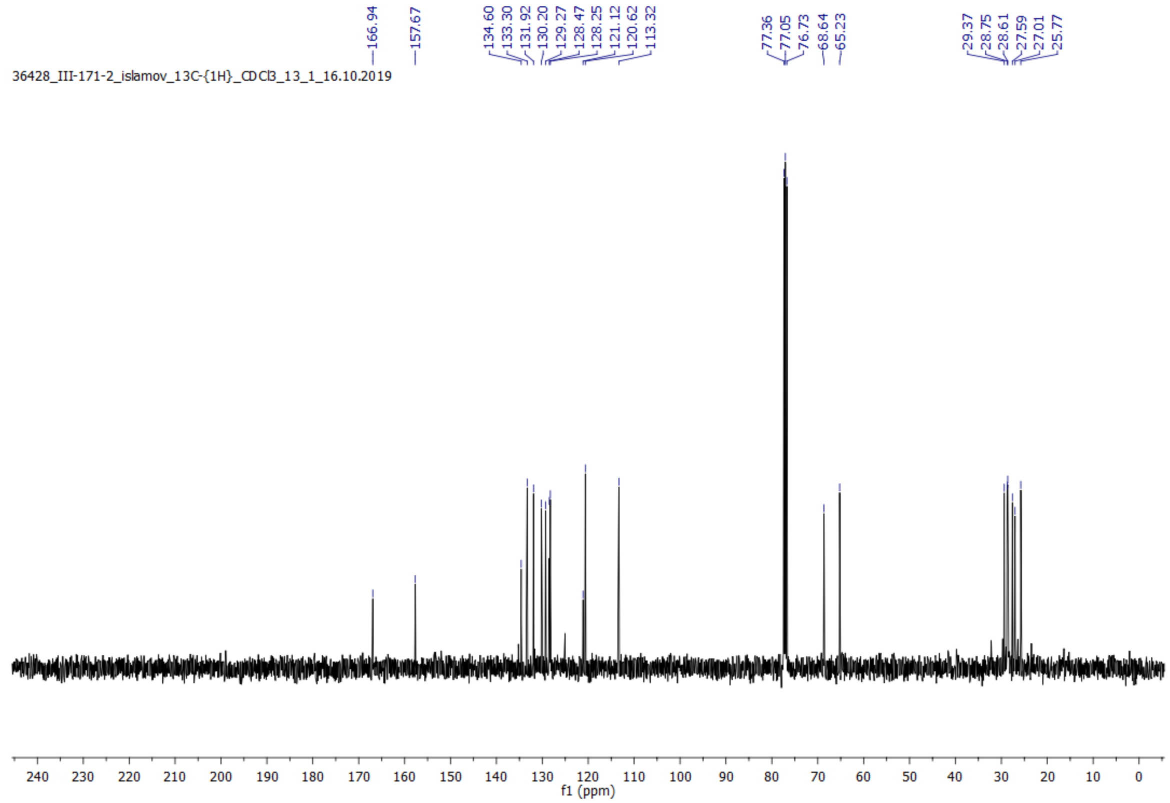
36428_III-171-2_islamov_13C-{1H}_CDCl3_13_1_16.10.2019
f1 (ppm)
